# Population Pharmacokinetic and Pharmacodynamic Study of Palbociclib in Children and Young Adults with Recurrent, Progressive, or Refractory Brain Tumors

**DOI:** 10.3390/pharmaceutics16121528

**Published:** 2024-11-28

**Authors:** John C. Panetta, Nicholas S. Selvo, David Van Mater, Clinton F. Stewart

**Affiliations:** 1 Department of Pharmacy and Pharmaceutical Sciences, St. Jude Children’s Research Hospital, 262 Danny Thomas Place, Memphis, TN 38105, USA; carl.panetta@stjude.org (J.C.P.); nick.selvo@stjude.org (N.S.S.); 2 Division of Pediatric Hematology-Oncology, Department of Pediatrics, Duke University Medical Center, Durham, NC 27710, USA; david.vanmater@duke.edu

**Keywords:** palbociclib, CDK4/6, pharmacokinetics, pharmacodynamics, pediatrics, brain tumors, special populations, PK/PD models, nonlinear mixed-effects modeling

## Abstract

**Background/Objectives:** Palbociclib, an oral CDK 4/6 inhibitor, was evaluated in a Pediatric Brain Tumor Consortium (PBTC) phase 1 (NCT02255461; PBTC-042) study to treat children and young adults with recurrent, progressive, or refractory brain tumors. The objectives of this study were to characterize the palbociclib population pharmacokinetics in children enrolled on PBTC-042, to conduct a population pharmacodynamic analysis in this patient population, and to perform a simulation study to assess the role of palbociclib exposure on neutropenia and thrombocytopenia. **Methods:** The palbociclib population pharmacokinetics and pharmacodynamics were characterized in this patient population (n = 34 patients; 4.9–21.6 years old). Population pharmacokinetics were modeled using a one-compartment model with first-order absorption and elimination. Covariate analysis was performed, evaluating demographics, laboratory values, and concomitant medications. A pharmacodynamic model was used to describe the relation between palbociclib plasma exposure and changes in the ANC and platelet counts. **Results:** The population estimates for the apparent oral volume, apparent oral clearance, and absorption rate constant were 664.5 L/m^2^, 36.8 L/h/m^2^, and 0.48 h^−1^, respectively. The palbociclib apparent oral clearance was decreased in patients with higher AST values (*p* = 0.0066). The ANC and platelet pharmacodynamic models estimated that the median (5th–95th percentile) time individuals had grade 3 or greater neutropenia was 4 (0, 21) days. Simulations showed that given 75 mg/m^2^ palbociclib, 49% of the individuals were expected to have grade 3 or greater neutropenia. **Conclusions:** Palbociclib pharmacokinetics and pharmacodynamics were adequately characterized in this patient population, no unexpected adverse reactions were noted, and the drug was well tolerated.

## 1. Introduction

Approximately 4000 new cases of pediatric central nervous system (CNS) tumors occur in the United States per year [1]. The current therapy for pediatric CNS tumors such as medulloblastomas, high-grade gliomas, and ependymomas includes maximal surgical resection and radiation alone or in combination with chemotherapy. Although this approach will lead to excellent outcomes in these tumors, some of these patients will recur or relapse, leading to poor overall survival. For these populations, alternative approaches including molecularly targeted agents are needed [2].

Recent studies at the genomic level have revealed numerous specific molecular alterations that drive the progression of these tumors, but these also may serve as the basis for the design of molecularly targeted drugs, which can control tumor proliferation and spread and, in contrast to cytotoxic therapy, cause less damage to normal tissues [3,4,5]. Specifically, studies have shown that a significant subset of pediatric CNS tumors harbor mutations in the cyclin-D cyclin-dependent kinase 4 and 6 (CDK4/6)-retinoblastoma (Rb) pathway that is a key regulator of the cell cycle. CDK4/6 inhibitors prevent progression from the G1 phase to the S phase of the cell cycle by inhibiting the phosphorylation of the retinoblastoma protein (RB1), thereby preventing replication [6,7].

Palbociclib is an oral, potent, and highly selective reversible inhibitor of cyclin-dependent kinases CDK4 and CDK6, which prevents RB1 phosphorylation and stops abnormal cellular replication. In children with recurrent or refractory solid or primary CNS tumors, the average time of the maximum observed concentration (T_max_) after oral palbociclib administration was between 5 and 8 h, and the average half-life was 11.3 to 19.5 h. The palbociclib systemic exposure (i.e., the maximum plasma concentration (C_max_) and area under the plasma concentration–time curve (AUC)) increased in a proportional manner; however, a large inter-individual variability in pharmacokinetic parameters was reported [8,9].

Myelosuppression, primarily neutropenia, has been the most common adverse event associated with palbociclib administration in both adult breast cancer studies and the pediatric literature [8,10]. This neutropenia is likely on-target since preclinical studies have shown that palbociclib inhibits neutrophil maturation through an effect on early myeloid progenitor cells [11]. The extent of neutropenia is associated with palbociclib systemic exposure in clinical trials in adults and in children with relapsed brain tumors; higher palbociclib systemic exposures (AUC and C_max_) were associated with severe (grade 3 or 4) neutropenia [8].

Although numerous adult population pharmacokinetic and pharmacodynamic studies have been published, no comprehensive analysis has been performed in children with cancer. Thus, the objectives of this study were to characterize the palbociclib population pharmacokinetics in children with recurrent, progressive, or refractory brain tumors enrolled on PBTC-042, to conduct a population pharmacodynamic analysis in this patient population, and to perform a simulation study to assess the role of palbociclib exposure on neutropenia and thrombocytopenia.

## 2. Patients and Methods

### 2.1. Clinical Trial Design

The patients included in this palbociclib population pharmacokinetic/pharmacodynamic analysis were treated in the phase I clinical trial PBTC-042 (NCT02255461), which evaluated single-agent oral palbociclib therapy in children and young adults with recurrent, progressive, or refractory brain tumors. Details of this clinical trial have been reported elsewhere [8]. In adults treated with palbociclib, myelosuppression has been the main dose-limiting toxicity (DLT) reported, and the toxicity is greater in more heavily pretreated patients [12,13]. Thus, patients in this pediatric trial were divided into two strata: stratum I included patients who were not heavily pretreated, while stratum II included heavily pretreated patients [8]. The starting dosage for stratum I was 50 mg/m^2^, with planned dose escalations to 75 mg/m^2^ and 95 mg/m^2^. The planned starting dosage for stratum II was one dosage level below the MTD for stratum I. The Institutional Review Board approved this trial and written informed consent was obtained from patients, parents, or legal guardians before the onset of any study procedures. Assent was obtained from patients as appropriate based on local institutional guidelines.

### 2.2. Pharmacokinetic Sampling and Bioanalysis

Pharmacokinetic studies of oral palbociclib (PD-0332991) were performed on course 1, day 1 and day 21 to evaluate the population pharmacokinetics of palbociclib. On day 2 of course 1, the palbociclib dose was held. On day 1 of course 1, palbociclib single-dose serial blood samples were drawn pre-dose and 0.5, 1, 2, 4, 8 (±1), 24 (±4), and 48 (±4) hours (immediately prior to the day 3 dose) after the oral dose. On day 21 of course 1, palbociclib steady-state serial blood samples were collected pre-dose and 1, 2, 4, 8 (±1), and 24 (±4) hours (immediately prior to the day 22 dose) after the dose.

At each time point, whole blood (2 mL) was collected in K_2_EDTA tubes, spun to plasma within one hour of collection, and stored at −20 °C until analysis. Palbociclib plasma concentrations were measured using a validated liquid chromatography–mass spectrometric assay method with a lower limit of quantitation (LLOQ) of 1 ng/mL [14].

### 2.3. Population Pharmacokinetic Analysis

Palbociclib serum concentration–time data from course 1, day 1 and day 21 were analyzed using nonlinear mixed-effects modeling implemented in Monolix software version 2023R1 (Lixoft, Antony, France) using the Stochastic Approximation Expectation Maximization (SAEM) algorithm. Inter-individual and inter-occasion variabilities were assumed to be log-normally distributed. A proportional residual error model was used to quantify the unexplained variability of the model. Error terms were assumed to be normally distributed. A one-compartment model with first-order absorption, the absorption lag time, and first-order elimination was used [15,16]. Specifically, the PK parameters estimated included the absorption lag time (T_lag_), absorption rate constant (k_a_), apparent oral volume (V/F), and apparent oral clearance (CL/F), where F was the bioavailability, which was not identifiable given only oral dosing was used. The inter-individual variability was estimated for all parameters. Additionally, the inter-occasion variability was estimated for the CL/F. The palbociclib dosage (i.e., the palbociclib dosage normalized to the patient body surface area (BSA)) was used as the model input. Data below the limit of quantitation (BLQ) were censored according to the Beal method M3 [17], as implemented in Monolix.

#### 2.3.1. Covariate Analysis

The following covariates were evaluated for their effects on the apparent oral palbociclib clearance: age, body weight, body surface area (BSA), gender, serum creatinine, serum albumin, estimated glomerular filtration rate (eGFR), aspartate aminotransferase (AST), total bilirubin, and concomitant medications reported in at least 10% of the total patients. The eGFR was calculated using a simplified two-covariate equation based on the patient height and serum creatinine, which was developed specifically for pediatric oncology patients [18]. Concomitant medications were tested as categorical variables (i.e., yes/no) and included dexamethasone, ranitidine, famotidine, granisetron, ondansetron, levetiracetam, lacosamide, lorazepam, levothyroxine, gabapentin, and trimethoprim/sulfamethoxazole. Continuous covariates were modeled using a power model scaled to the population median covariate value. Categorical covariates were modeled according to an exponential model.

A stepwise forward inclusion method was used to identify significant covariates. Each covariate was added univariately to the parameter of interest and selected based upon a decrease in the OFV by at least 3.84 units (corresponding to *p* < 0.05 based on the χ^2^ test), a decrease in the inter-individual variability, and improved model fits. The covariate displaying the highest change in the OFV was retained in the model and the process was repeated until no covariates were statistically significant.

#### 2.3.2. Population Pharmacokinetic Model Evaluation

The population pharmacokinetic model fit was evaluated using various goodness-of-fit plots including observed vs. population predicted, observed vs. individual predicted, weighted residuals vs. time, and visual predictive checks. The predictive performance of the final model was evaluated using approaches previously employed [19,20]. The precision of the parameters estimated from the final population model was evaluated using a non-parametric bootstrap resampling method. A total of 500 replicates of the original dataset were generated using random sampling with replacement. The model was applied to each replicate dataset. Summary statistics for each parameter were calculated based upon the model runs (i.e., the mean and 90% bootstrap confidence interval). Non-parametric bootstrapping was performed with the MonolixSuite R-based package Rsmlx 2.0.4 package (Lixoft, Antony, France).

### 2.4. Pharmacodynamic (PD) Methods

A complete blood count (CBC) with differential was performed pre-therapy, weekly during course 1, and then weekly during courses 2 to 26. The CBC provided platelet and absolute neutrophil counts (ANCs). If an individual only had a single platelet count or ANC observation collected before palbociclib administration, the patient was not included in the pharmacodynamic analysis.

#### 2.4.1. Pharmacodynamic Model

The model describing the ANC and platelet dynamics has been previously described [21]. The model for the ANC consisted of a bone marrow compartment with proliferating neutrophils, three transient compartments that represent the maturation process of the neutrophils from blasts to circulating neutrophils, and a circulating neutrophil compartment. The model included the negative feedback effects of endogenous G-CSF, which affected the growth rate of proliferating cells in a manner inversely proportional to the concentration of circulating neutrophils. We assumed that palbociclib only affected the proliferating neutrophils in the bone marrow compartment. We used a similar structural model for platelet dynamics. The model describing the neutrophil dynamics is shown in Equations (1)–(5) and Figure S1.

(1) $$\frac{N_{PBM}}{dt}=\left[\frac{k_{in}K_{M}}{K_{M}+N_{circ}}\frac{IC_{50}^{n}}{IC_{50}^{n}+C_{palblociclib}^{n}}-k_{bp}\right]N_{PBM}$$

(2) $$\frac{dN_{T1}}{dt}=k_{bp}\left(N_{PBM}-N_{T1}\right)$$

(3) $$\frac{dN_{T2}}{dt}=k_{bp}\left(N_{T1}-N_{T2}\right)$$

(4) $$\frac{dN_{T3}}{dt}=k_{bp}\left(N_{T2}-N_{T3}\right)$$

(5) $$\frac{dN_{circ}}{dt}=kpbN_{T3}-k_{out}N_{circ}$$

The model parameters were as follows: *N_PBM_*: proliferating bone marrow stem cells; *N_T_*_1_, *N_T_*_2_, and *N_T_*_3_: transient compartments describing the maturation phases of the neutrophils; *N_circ_*: circulating neutrophils. Note that we were only able to observe this compartment. The model parameters were defined as follows: *k_in_* (1/days): the stem cell proliferation rate; *k_bp_* (1/days): the transition rate between transient compartments (the mean transition time from the bone marrow to the circulating neutrophils is 4/*k_bp_*); *k_out_* (1/days): the elimination rate of circulating neutrophils; *K_M_* (10^3^/µL): the half-saturation effect of the G-CSF negative feedback; *IC*_50_ (nM) and *n*; the half-saturation effect and Hill coefficient of the palbociclib effects on the bone marrow; and *C_palbociclib_* (nM): the concentration of palbociclib in the plasma. Prior to treatment, the system was assumed to be steady-state, where *N_circ_*_0_ was the steady-state circulating neutrophil level. The following relationship between the model parameters was needed for the system to maintain steady state prior to treatment (Equation (6)).

(6) $$K_{M}=\frac{k_{bp}}{k_{in}-k_{bp}}N_{circ0}$$

For this relationship to be positive, *k_in_* > *k_pb_*. To ensure this requirement, we defined *k_pb_ = fk_bp_·k_in_* where *fk_bp_* ∈ [0, 1] and estimated *fk_bp_*. In addition, the initial conditions for the system at steady state (i.e., the steady-state solution to the system in the absence of the drug) are (Equation (7))

(7) $$\left[\frac{k_{out}}{k_{bp}}N_{circ0},\;\frac{k_{out}}{k_{bp}}N_{circ0},\;\frac{k_{out}}{k_{bp}}N_{circ0},\;\frac{k_{out}}{k_{bp}}N_{circ0},\;N_{circ0}\right]$$

#### 2.4.2. Parameter Estimation

The parameters for the pharmacodynamic model were estimated using nonlinear mixed-effects modeling with the SAEM algorithm as implemented in the Monolix^®^ software version 2023R1 (Lixoft, Antony, France). The palbociclib pharmacokinetics were fixed to each individual’s post hoc estimated parameters (as determined above). The inter-individual and inter-occasion variability were assumed to be log-normally distributed for all parameters except *fk_bp_*, which was assumed to be a logit distribution, so its value will be between 0 and 1. A proportional residual error model was used to quantify the unexplained variability of the model. Error terms were assumed to be normally distributed.

#### 2.4.3. Pharmacodynamic Model Simulations

To assess the extent of neutropenia and thrombocytopenia expected at clinically relevant dosages and the effect of prior myelosuppressive therapy on neutropenia and thrombocytopenia, simulations were performed with three fixed dosages (50, 75, or 95 mg/m^2^/day for 21 days) using the individual estimated PK (conditional mode) and PD parameters (samples from the conditional distribution). The median and 5th–95th percentiles for the measurements of neutropenia and thrombocytopenia were calculated using the individual PD profiles (based on the individual estimated PD parameters sampled from the individual conditional distribution) of the ANC and platelet dynamics. We evaluated bias in residuals (measured vs. estimated ANC or platelets) between courses (1 and 2 vs. 3 and 4) using the Kruskal–Wallis test. Comparisons of neutropenia and thrombocytopenia between strata were evaluated using the Kruskal–Wallis test.

## 3. Results

### 3.1. Summary of Population Pharmacokinetic Data

Pharmacokinetic studies were conducted on the 34 participants enrolled in the study who were assigned oral palbociclib at 50, 75, and 95 mg/m^2^/day. One patient who deviated from the protocol by taking concomitant medications of phenytoin (a strong CYP3A inducer) and omeprazole (a proton pump inhibitor) was excluded from the analysis. The remaining 33 patients participated in course 1, day 1 pharmacokinetic studies, and of them, 26 patients participated in course 1, day 21 studies. Among the seven patients who did not participate in course 1, day 21 pharmacokinetic studies, one patient withdrew consent, four patients went off study, one patient was taken off treatment due to progressive disease, and samples from one patient were not collected because of that patient’s clinical condition. Patients ranged from 4.9 to 21.6 years old, and over half were Caucasian. All the patient characteristics, including the clinical covariates and concomitant medications of interest, are summarized in Table 1.

This study included 397 samples from these 33 individuals. We excluded 10 samples from three patients, so a total of 387 samples were included in the pharmacokinetic analysis. The 10 samples were excluded for the following reasons: the 24 h sample was higher than the 8 h in two individuals, suggesting that these patients may have taken a dose prior to the sample at 24 h. Therefore, the 24 and following 48 h samples in these two individuals were excluded. One patient had four course 1, day 21 samples that were BLQ (pre-dose and 1, 2, and 4 h post-dose), followed by two samples well above BLQ at 7 and 24 h post-dose. These samples BLQ were spurious given the individual received 21 days of dosing. Therefore, these six samples on day 21 were excluded.

### 3.2. Population Pharmacokinetic and Covariate Analysis

**Figure 1:**
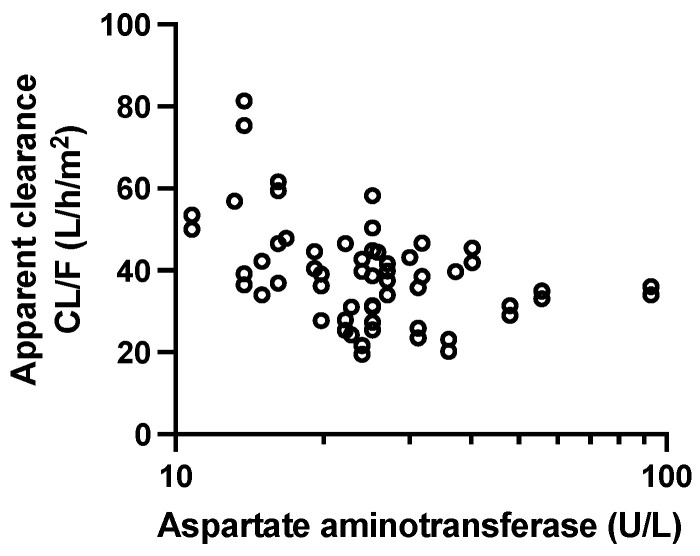
Scatterplot of palbociclib apparent clearance and aspartate aminotransferase levels (Corr: 0.98, *p* = 0.0066).

The base population pharmacokinetic estimates for the T_lag_, k_a_, V/F, and CL/F were 0.8 h, 0.48/h, 664.5 L/m^2^, and 36.8 L/h/m^2^, respectively. A summary of all the population parameters and measures of variability is shown in Table 2. Covariate analysis showed that the palbociclib CL/F was significantly decreased with increasing AST (from 48.2 L/h/m^2^ at AST = 10 U/L to 24.1 L/h/m^2^ at AST = 100 U/L; *p* = 0.0066; Table S1; Figure 1). With the inclusion of AST, the inter-patient variability of the palbociclib CL/F decreased by 10% (from 0.29 to 0.26) (Table 2). None of the concomitant drugs considered as covariates were significant (Table S1).

**Figure 2:**
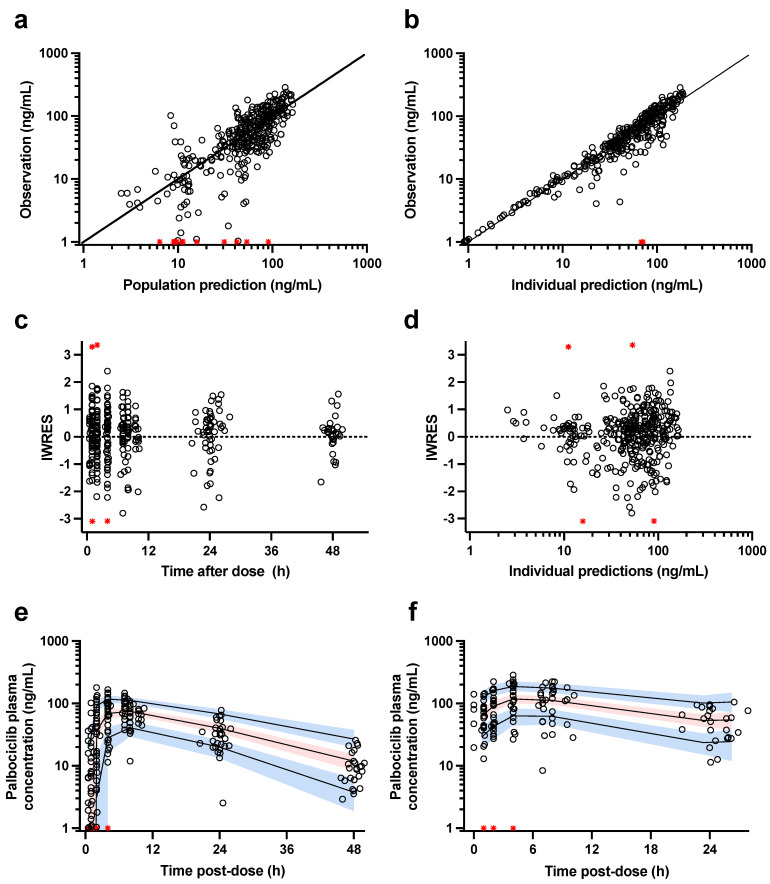
Diagnostic plots for the population pharmacokinetic model. (**a**) Observed concentration–time data vs. population, (**b**) individual model predictions, (**c**) individual weighted residuals (IWRES) vs. time, and (**d**) model predictions. In panels (**a**,**b**), solid lines represent the unity line and red crosses are data below the limit of quantification. The bottom plots show (**e**) the prediction-corrected visual predictive checks for single-dose and (**f**) steady-state data. Circles are observed data, stars in a red color are data below the limit of quantification, the solid lines depict the model-predicted 5th, 50th, and 95th percentiles, respectively, and the shaded areas represent the 90th confidence interval around the model-predicted percentiles.

The diagnostic plots of the final pharmacokinetic model are shown in Figure 2. The goodness-of-fit plots based on the model observations and predictions did not show any significant bias or model misfits (Figure 2a–d). In addition, the prediction-corrected visual predictive checks based on model simulations showed that the central tendency and the variability of the palbociclib data both after a single dose and at steady state were accurately described by the final population pharmacokinetic model (Figure 2e,f).

The precision of the population parameter estimates for the final model was further assessed using non-parametric bootstraps. The estimated population parameters were close to the mean of the bootstrap estimates, and none of the 90% confidence intervals included a value of zero (Table 2).

### 3.3. Summary of Pharmacodynamic Data

Of the 33 patients used for the population PK model, 2 individuals were not included in the pharmacodynamic analysis as they only had a single ANC and PLT observation collected before the administration of the palbociclib dose. A total of 190 ANCs and 189 PLT observations from 31 individuals were available during course 1 (until the beginning of course 2, day 1). A total of 99 ANCs and PLT observations from 21 individuals were available during course 2 (until the beginning of course 3, day 1). A total of 54 ANCs and 55 PLT observations from six individuals were available during courses 3 and 4. An average of 11 ANC and PLT data points were collected for each patient over all courses.

### 3.4. Population Pharmacodynamic Parameter Estimation—ANC

**Figure 3:**
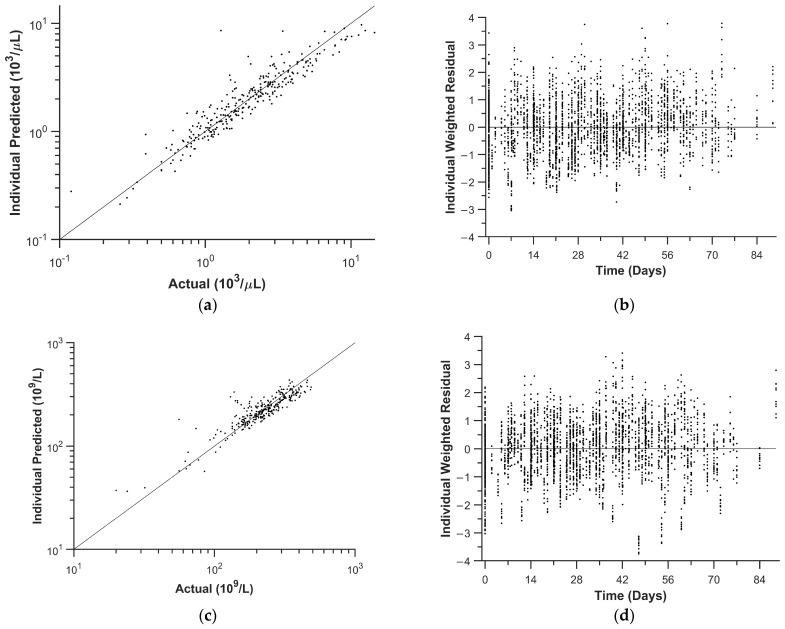
Pharmacodynamic model goodness-of-fit plots for ANC: (**a**) Actual vs. individual predicted ANC. (**b**) Individual weighted residuals for ANC. Pharmacodynamic model goodness-of-fit plots for PLT: (**c**) Actual vs. individual predicted PLT. (**d**) Individual weighted residuals for PLT.

We used the ANC data for the first two courses of palbociclib to evaluate the population pharmacodynamic parameters. A summary of the population pharmacodynamic parameters is shown in Table 3A, the goodness-of-fit plots are shown in Figure 3a,b, and individual ANC vs. time plots of the model fits are shown in Figure S2. Based on the individual model estimates, the estimated median (5th–95th percentile) time below an ANC of 1.5 × 10^3^/µL (i.e., grade 2 neutropenia), 1.0 × 10^3^/µL (i.e., grade 3 neutropenia), and 0.5 × 10^3^/µL (i.e., grade 4 neutropenia) was 15 (0, 35), 4 (0, 21), and 0 (0, 11) days/course for the first two courses, respectively. In addition, the estimated median (5th–95th percentile) nadir based on data from the first two courses was 1.0 (0.4, 3.3) × 10^3^/µL.

Using the six individuals who also had course 3 and 4 ANC data, we determined the ability to predict these next two courses of treatment given the PKs from course 1 and the PD estimates based on data from courses 1 and 2. Figure S2 shows the individual ANC vs. time plots for all four courses for these six individuals, visually indicating that courses 3 and 4 are described well by the individual model fits to the first two courses. Additionally, we evaluated the residuals (the percent difference between the observed and estimated ANC) during courses 1 and 2 vs. 3 and 4. We observed no bias in the residuals in either group (median [5th–95th percentile]: −1.5% [−41%, 38%], *p* = 0.16, and −6.1% [−69%, 55%], *p* = 0.22, respectively), and the difference in the bias between the two groups was not significant (*p* = 0.1).

### 3.5. Population Pharmacodynamic Parameter Estimation—Platelets

We then used the PLT data for the first two courses of palbociclib to evaluate the population pharmacodynamic parameters. A summary of the population parameters is shown in Table 3B, goodness-of-fit plots are shown in Figure 3c,d, and individual PLT vs. time plots of the model fits are shown in Figure S3. Based on the individual model estimates, only three individuals had grade 1 (<100 × 10^9^/μL) or greater thrombocytopenia (one grade 1, one grade 2, and one grade 3). In addition, the estimated median (5th–95th percentile) nadir was 169 (59, 240) × 10^9^/μL.

Using the six patients who also had course 3 and 4 PLT data, we determined the ability to predict these next two courses of treatment given the PKs from course 1 and the PD estimates based on data from courses 1 and 2. Figure S3 shows the individual PLT vs. time plots for all four courses in these six individuals, visually indicating that courses 3 and 4 are described well by the individual model fits to the first two courses. Additionally, we evaluated the residuals (the percent difference between the observed and estimated PLT) during courses 1 and 2 vs. 3 and 4. We observed no bias in the residuals in courses 1 and 2 (median [5th–95th percentile]: −1.9% [−26%, 28%], *p* = 0.08), though there was some bias (over-predicting the PLT depletion) in courses 3 and 4 (−8.0% [−37%, 18%], *p* = 4 × 10^−4^). The difference in the bias between the two groups was also significant (*p* = 0.006).

### 3.6. Pharmacodynamic Simulations

**Figure 4:**
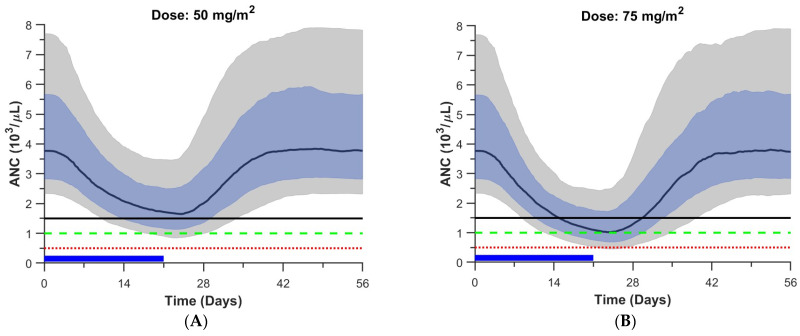

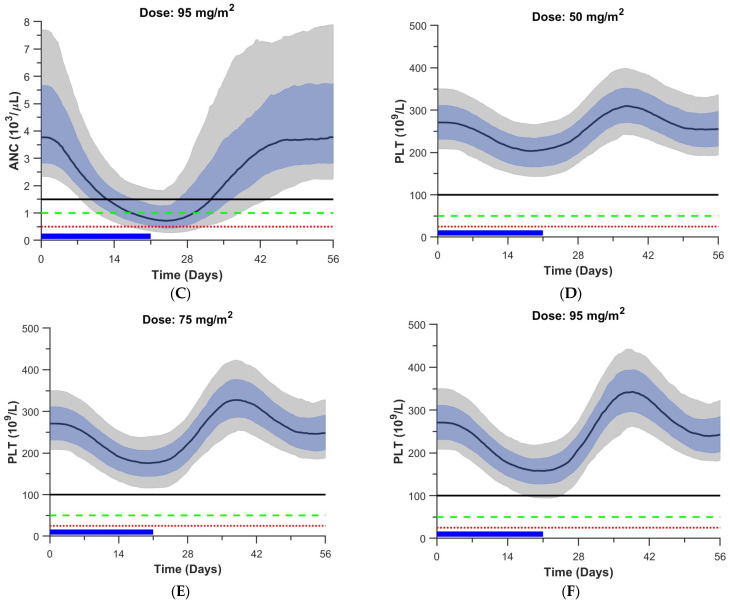
Pharmacodynamic simulations: (**A**): Simulated ANC vs. time for 50 mg/m^2^/day palbociclib for 21 days. (**B**): Simulated ANC vs. time for 75 mg/m^2^/day palbociclib for 21 days. (**C**): Simulated ANC vs. time for 95 mg/m^2^/day palbociclib for 21 days. (**D**): Simulated PLT vs. time for 50 mg/m^2^/day palbociclib for 21 days. (**E**): Simulated PLT vs. time for 75 mg/m^2^/day palbociclib for 21 days. (**F**): Simulated PLT vs. time for 95 mg/m^2^/day palbociclib for 21 days. For all panels of Figure 4—blue bar: dosing interval; black solid line, green dashed line, and red dotted line: mild neutropenia or thrombocytopenia (100 × 10^9^/L), moderate neutropenia (1.0 × 10^3^/µL) or thrombocytopenia (50 × 10^9^/L), and severe neutropenia (0.5 × 10^3^/µL) or thrombocytopenia (25 × 10^9^/L); black curve: median ANC or PLT; blue shaded region: 25th–75th percentiles; gray shaded region: 5th–95th percentiles.

To assess the extent of myelosuppression expected at clinically relevant dosages, we simulated the effects of a fixed palbociclib dosage of either 50, 75, or 95 mg/m^2^/day for 21 days on the duration and extent of ANC and PLT depletion. In these simulations, we used PD parameters sampled from each patient’s conditional distribution (n = 10 per individual). These results are summarized in Figure 4 and Table 4. These simulations show that given a 50 mg/m^2^ dose, 19% of the individuals are expected to have grade 3 or greater neutropenia and none are expected to have grade 3 or greater thrombocytopenia. Furthermore, the median (5th–95th percentile) duration of grade 3 or greater neutropenia was 0 (0, 16) and 12 (0, 30) days for a 50 and 95 mg/m^2^ dose, respectively. In addition, given a 95 mg/m^2^ dose, 69% of the individuals are expected to have grade 3 or greater neutropenia and 6% of the individuals are expected to have grade 3 or greater thrombocytopenia, and the median (5th–95th percentile) duration of grade 3 or greater thrombocytopenia was 0 (0, 0) and 0 (0, 11) days for a 50 and 95 mg/m^2^ dose, respectively.

To assess the effect of prior myelosuppressive therapy on myelosuppression, we simulated patients subdivided by stratum, and our simulations show that given a dosage of 75 mg/m^2^, more grade 3 neutropenia would be expected in a heavily pretreated patient (i.e., stratum 2) than a patient who was not heavily pretreated (i.e., stratum 1) (75% vs. 35%; *p* = 2 × 10^−18^; Figure S4) and more grade 3 thrombocytopenia would be expected in stratum 2 vs. 1 (15% vs. 0%; *p* = 3 × 10^−13^; Figure S5).

## 4. Discussion

The population pharmacokinetics and pharmacodynamics of palbociclib, a CDK4/6 inhibitor, were characterized for the first time in children and young adults with recurrent, progressive, and refractory brain tumors. The results of the covariate analysis showed that the apparent oral clearance of palbociclib was influenced by baseline AST values. Specifically, a 2.5-fold increase in the baseline AST was associated with a 2-fold lower apparent oral palbociclib clearance. Additionally, the ANC and PLT data during the first two courses were well described. Based on the individual model estimates for the ANC and PLT for the first two courses, we predicted the ANC data for six patients for courses 3 and 4 without bias, but the PLT data were biased and slightly over-predicted.

Prior to the current report, only one study had published pharmacokinetic data for palbociclib in pediatric oncology patients. Raetz and colleagues studied the disposition of palbociclib in 12 children with acute lymphoblastic leukemia or lymphoma enrolled in a phase I study [22]. They obtained serial samples on days 1 and 11 for up to 24 h after the oral dose. Both capsule (n = 3) and liquid formulations (n = 9) were used in this study, and a noncompartmental pharmacokinetic analysis approach was used. They reported a mean (range) steady state apparent oral clearance of 40.4 (21.1–102.0) L/h/m^2^ and a half-life of 27.9 (4.5–86.8) hours [22].

In adult patients, the results of several population pharmacokinetic studies have been published. Royer and colleagues published the first population pharmacokinetic study of palbociclib utilizing retrospective data collected from therapeutic drug monitoring [15]. Although they studied 124 patients, they only had 151 samples for model building. A one-compartment model with a first-order absorption (Ka), an absorption lag time, and a combined error model was used to describe their data. The noise in the real-world data limited their ability to estimate the parameters of their model (e.g., the absorption); however, their estimate of a palbociclib apparent oral clearance of 58 L/h (34 L/h/m^2^) compares well with the one from the present study. They found that the creatinine clearance (CrCl) estimated using the Cockcroft–Gault method, which includes the variables of age, gender, weight, and serum creatinine, was a significant covariate. As the CrCl decreased, the palbociclib apparent oral clearance decreased, and the AUC increased.

That same group conducted a population pharmacokinetic/pharmacodynamic study of palbociclib using therapeutic drug monitoring data for 143 patients, which were retrieved retrospectively from the patient record management software [16]. Similarly to Royer, these authors used a one-compartment model with a first-order absorption (Ka), an absorption lag time, and a combined error model to describe their data. They estimated the palbociclib apparent oral clearance as 57 L/h (34 L/h/m^2^), which is remarkably similar to that of Royer and not unexpectedly compares to the one from the present study. As with the study by Royer, these authors also found that CrCl was a significant covariate for the palbociclib apparent oral clearance. However, these authors also found that a hepatic biomarker (alkaline phosphatase; ALP) was a significant covariate for the palbociclib apparent oral clearance with increasing ALP associated with a decreasing palbociclib clearance and increased systemic exposure.

Lastly, Courlet and colleagues conducted a population pharmacokinetic study in adults using data from a clinical trial aimed at optimizing oral targeted anticancer therapies (OpTAT; NCT04484064) [23]. The authors obtained blood samples at routine visits and serial sampling up to 12 h in 45 women with metastatic breast cancer receiving palbociclib. Differently from the Royer analysis, these authors used a two-compartment model with first-order absorption, a lag-time, and a proportional error model to describe their data. The palbociclib apparent oral clearance from their analysis was 67 L/h (39 L/h/m^2^), which was similar to both Royer and the current analysis. In this analysis, the palbociclib apparent oral clearance was increased by 56% when taken under fasting conditions along with concomitant proton pump inhibitors; however, the authors never specify exactly which PPIs these patients were administered nor the exact palbociclib formulation used.

The results of our covariate analysis showed that aspartate aminotransferase (AST) was a significant covariate for the palbociclib apparent oral clearance. Our covariate analysis included a measure of renal function (i.e., the estimated GFR) as well as age and BW, but none of these covariates had a significant impact on the palbociclib apparent oral clearance as found by Royer or Marouille in their analyses [15,16]. We did find that a marker of hepatic function, AST, was associated with the palbociclib apparent oral clearance, similarly to that of Royer. Moreover, as reported by those authors, an increase in AST was associated with a decrease in the palbociclib apparent oral clearance and an increase in the palbociclib systemic exposure.

The results of the first phase I studies in adults showed that myelosuppression was the primary dose-limiting toxicity for palbociclib. Thus, investigators performed exploratory pharmacokinetic and pharmacodynamic analyses of the relation between palbociclib systemic exposure and the ANC and platelet levels during the first two courses of treatment [24,25]. Using a simple Emax model, they established a relationship between the change in the ANC and platelet levels versus the palbociclib plasma exposure with increasing exposures resulting in a saturable decrease from the baseline for both the ANC and platelets. Subsequent studies used a more mechanistic population pharmacokinetic/pharmacodynamic modeling approach based on a previously described semi-mechanistic physiological model developed to describe the effects of cytotoxic chemotherapy agents on neutrophils but not platelets [26]. Although the Friberg approach initially was intended to model the neutropenic effects of cytotoxic chemotherapy, these investigators found that it performed well at modeling the cytostatic effects of palbociclib [16,27,28]. This model has also been used to model the cytostatic effects of other CDK4/6 inhibitors such as ribociclib [29] and trilaciclib [30].

Similarly to the results observed in the adult phase I studies, the recent pediatric phase I study of palbociclib also reported myelosuppression as the most common dose-limiting toxicity [8]. A preliminary regression analysis demonstrated that higher values of the palbociclib Cmax and AUC were associated with more severe (grade 3 or 4) neutropenia and leukopenia [8]. These observations prompted this more thorough analysis of the relation between palbociclib systemic exposure and the ANC and platelet levels. We modeled both the ANC and PLT data in the current study using our previously developed mechanistic mathematical model originally developed to quantify temozolomide myelosuppression [31] and later modified to quantify topotecan neutropenia and thrombocytopenia in neuroblastoma [21]. The results of our current analysis compare closely to several other studies. For example, the baseline ANC (CIRC_0_) for our model was 3.95 × 10^3^/µL, which compares favorably with 3.63 × 10^3^/µL from Sun [27], 2.94 × 10^3^/µL from Marouille [16], and 3.36 × 10^3^/µL from Jian [28]. In addition, the MTT (the time for committed stem cells to pass through the maturation compartments before entering the circulation) in the three published adult PK/PD studies ranged from 4.33 to 8.25 days, which was similar to the 5.12 days estimated from our current study. 

Since six patients had ANC data from the third and fourth courses, we determined the ability of our pharmacodynamic model to use course 1 and 2 individual PD and PK estimates to predict the PD profile for courses 3 and 4. The lack of bias in the residuals indicated that our model was capable of predicting course 3 and 4 ANC values well and that the decreases in ANCs were not cumulative. This observation was consistent with that made in the early phase I study of Schwartz and colleagues [24]. In a larger population PK/PD analysis, Sun and his group also noted neutropenia associated with palbociclib administration was reversible and not cumulative [27].

Using our pharmacodynamic model, we simulated the extent of myelosuppression expected at clinically relevant palbociclib dosages (between 50 and 95 mg/m^2^). The simulations provide a quantitative and mechanistic approach to predict the ANC and PLT concentrations expected at any clinically relevant palbociclib dosage. For example, the model predicted that given a palbociclib dosage of 75 mg/m^2^, approximately half the patients would have grade 3 or greater neutropenia.

The present study had several limitations. First, as with many pediatric population pharmacokinetic and pharmacodynamic studies, the number of patients studied was relatively small on course 1, day 1, and the number of patients with repeat studies on course 1, day 21 was even smaller. Another limitation of our study is the lack of external model validation. Due to the small patient population, the sample size was not large enough to derive an external validation group to further validate the model. Regardless, our pharmacokinetic and pharmacodynamic findings for this patient population agree with previously published adult results, and our PK/PD model could be very useful for predicting expected ANC- and PLT-related toxicities at relevant doses with the caveat that it will require prospective validation in an independent patient population.

## 5. Conclusions

This is the first population pharmacokinetic and pharmacodynamic study of palbociclib in children and adolescents with progressive brain tumors. Significant covariates associated with palbociclib disposition identified in our analysis included a decreased apparent oral clearance in patients with increased AST values. The ANC and PLT data during the first two courses were well described. Based on their individual model estimates for the ANC and PLT for the first two courses, we estimated the ANC data for six patients for courses 3 and 4 without bias, but the PLT data were biased and slightly over-predicted.

## Figures and Tables

**Table 1:** Characteristics of patients included in the pharmacokinetic study (n = 33).

Female/male n (%)	12 (36)/21 (64)
^†^ Race n (%)	
Caucasian	19 (58)
Black	4 (12)
Other	10 (30)
Stratum I/II n (%)	21 (64)/12 (36)
Palbociclib dosage n (%)	
50 mg/m^2^	6 (18)
75 mg/m^2^	21 (64)
95 mg/m^2^	6 (18)
Age (y)	12.8 (4.9–21.6)
Height (cm)	147.8 (109–185.1)
Weight (kg)	52.5 (23.8–110.4)
Body surface area (m^2^)	1.5 (0.8–2.4)
Albumin (g/dL)	4.2 (3.2–4.9)
Aspartate aminotransferase (AST; U/L)	25 (11–93)
Bilirubin (mg/dL)	0.4 (0.1–1.2)
Creatinine (mg/dL)	0.5 (0.2–1.2)
Concomitant medication: Day 1 n (%); Day 21 n (%)	
Ranitidine	3 (9); 2 (8)
Famotidine	1(3); 1(4)
HT-3 inhibitors (ondansetron/granisetron)	5 (15)/2 (6); 3 (12)/2 (8)
Dexamethasone	8 (24); 5 (19)

Data are reported as the frequency or median (range). ^†^ Self-declared ethnicity. Other includes Asian (n = 3), Hispanic (4), American Indian/Alaskan (1), and unknown (n = 2).

**Table 2:** Final pharmacokinetic population parameter estimates.

Parameters *	Base Model Est. (RSE%) *			Non-Parametric Bootstrap
		AST	Mean	90% CI
**Population estimates**				
Absorption lag time T_lag_ (h)	0.8 (12.3)	0.8 (12.1)	0.81	0.65; 0.97
Absorption rate constant k_a_ (/h)	0.48 (17.9)	0.46 (17.6)	0.51	0.39; 0.70
Volume V/F (L/m^2^)	664.5 (4.1)	653 (3.5)	668.5	617.1; 757.1
Clearance CL/F (L/h/m^2^)	36.8 (5.8)	36.5 (5.2)	33.5	30.5; 37.9
**Covariates**				
AST on CL/F		−0.3 (39.3)	−0.24	−0.12; −0.36
Inter-patient variabilities (IIV) *				
Absorption lag time T_lag_	0.68 (14.4)	0.67 (14.1)	0.69	0.54; 0.88
Absorption rate constant k_a_	0.88 (16)	0.87 (14.8)	0.86	0.34; 1.19
Volume V/F	0.076 (58)	0.04 (86)	0.06	0.02; 0.26
Clearance CL/F	0.29 (16.7)	0.26 (16.3)	0.24	0.15; 0.30
**Inter-occasion variabilities (IOV)**				
Clearance CL/F	0.13 (28)	0.12 (25.4)	0.13	0.09; 0.18
Residual proportional error	0.32 (4.7)	0.32 (4.7)	0.32	0.26; 0.39
2 × Log-likelihood value (OFV)	3198	3191.6		

* RSE%, relative standard error; IIV and IOV are reported as a standard deviation.

**Table 3:** (**A**) Population PD parameter estimates for ANC model. (**B**) Population PD parameter estimates for PLT model.

(A)						
Parameter	Population Estimate	RSE (%)	IIV (CV%)	RSE (%)	IOV (CV%)	RSE (%)
N_circ0_ (10^3^/µL)	3.95	8.7	47.0	13.9		
k_in_ (1/days)	0.90	8.6	29.4	50.2	14.3	54.3
k_bp_ (1/days)	0.78	3.5	1.5	203.4	5.8	123.7
k_out_ (1/days)	4.80	Fixed	Fixed			
IC_50_ (nM)	383.5	17.8	25.6	53.0	24.2	149.4
n	1	Fixed	Fixed			
**Residual error**						
b	0.29	6.0				
**(B)**						
**Parameter**	**Population Estimate**	**RSE** **(%)**	**IIV** **(CV%)**	**RSE** **(%)**	**IOV** **(CV%)**	**RSE** **(%)**
N_circ0_ (10^9^/L)	267.40	4.2	21.4	14.4		
k_in_ (1/days)	0.93	3.3	7.6	39.4	4.1	43.8
k_bp_ (1/days)	0.52	5.9	7.6	99.1	5.9	57.2
k_out_ (1/days)	4.80	Fixed	Fixed			
IC_50_ (nM)	559.14	11.2	33.0	33.6	19.6	72.8
n	1	Fixed	Fixed			
**Residual error**						
b	0.20	6.0				

**Table 4:** Estimated nadir and duration of ANC and PLT depletion when given palbociclib 50, 75, or 95 mg/m^2^/day for 21 days.

**Nadir of ANC**	**50 mg/m^2^/day**	**75 mg/m^2^/day**	**95 mg/m^2^/day**
% <1.5 × 10^3^/µL	45	72	79
% <1.0 × 10^3^/µL	19	49	69
% <0.5 × 10^3^/µL	3.1	13	32
**Duration of ANC depletion (days); median (5th–95th percentile)**			
Days < 1.5 × 10^3^/µL	0 (0, 28)	15 (0, 34)	20 (0, 39)
Days < 1.0 × 10^3^/µL	0 (0, 16)	0 (0, 24)	12 (0, 30)
Days < 0.5 × 10^3^/µL	0 (0, 0)	0 (0, 11)	0 (0, 19)
**Nadir of PLT**	**50 mg/m^2^/day**	**75 mg/m^2^/day**	**95 mg/m^2^/day**
% <100 × 10^9^/L	6	8	12
% <50 × 10^9^/L	0	6	6
% <25 × 10^9^/L	0	0	3
**Duration of PLT depletion (days); median (5th–95th percentile)**			
Days < 100 × 10^9^/L	0 (0, 1)	0 (0, 13)	0 (0, 16)
Days < 50 × 10^9^/L	0 (0, 0)	0 (0, 6)	0 (0, 11)
Days < 25 × 10^9^/L	0 (0, 0)	0 (0, 0)	0 (0, 0)

## Data Availability

Any requests for data from this study must be made in writing to the PBTC Operations, Biostatistics, and Data Management Core Executive Director.

## Supplementary Materials

The following supporting information can be downloaded at: https://www.mdpi.com/article/10.3390/pharmaceutics16121528/s1, Table S1: Comparison of the base pharmacokinetic model’s objective function value (OFV) with models including single covariates on apparent clearance (Cl/F); Figure S1: Depicted below is the pharmacodynamic model scheme describing the ANC and platelet dynamics. The model for ANC consists of a bone marrow compartment with proliferating neutrophils, three transient compartments that represent the maturation process of the neutrophils from blasts to circulating neutrophils, and a circulating neutrophil compartment. The model included the negative feedback effects of endogenous G-CSF, which affected the growth rate of proliferating cells in a manner inversely proportional to the concentration of circulating neutrophils. We assumed that palbociclib only affected the proliferating neutrophils in the bone marrow compartment. We used a similar structural model for platelet dynamics; Figure S2_1: ANC vs Time Plots for patient #1. Right Axis: ANC (10^3^/µL), black curve, model estimated curve; black dots measured ANC levels. Left Axis: palbociclib concentration (nM), red dotted curve, model estimated concentration. Horizontal black solid, green dashed, and red dotted lines: ANC thresholds of 1.5, 1.0, and 0.5 × 10^3^/µL, respectively; Figure S2_5: ANC vs Time Plots for patient #5. Right Axis: ANC (10^3^/µL), black curve, model estimated curve; black dots measured ANC levels. Left Axis: palbociclib concentration (nM), red dotted curve, model estimated concentration. Horizontal black solid, green dashed, and red dotted lines: ANC thresholds of 1.5, 1.0, and 0.5 × 10^3^/µL, respectively; Figure S2_14: ANC vs Time Plots for patient #14. Right Axis: ANC (10^3^/µL), black curve, model estimated curve; black dots measured ANC levels. Left Axis: palbociclib concentration (nM), red dotted curve, model estimated concentration. Horizontal black solid, green dashed, and red dotted lines: ANC thresholds of 1.5, 1.0, and 0.5 × 10^3^/µL, respectively; Figure S2_21: ANC vs Time Plots for patient #21. Right Axis: ANC (10^3^/µL), black curve, model estimated curve; black dots measured ANC levels. Left Axis: palbociclib concentration (nM), red dotted curve, model estimated concentration. Horizontal black solid, green dashed, and red dotted lines: ANC thresholds of 1.5, 1.0, and 0.5 × 10^3^/µL, respectively; Figure S2_22: ANC vs Time Plots for patient #22. Right Axis: ANC (10^3^/µL), black curve, model estimated curve; black dots measured ANC levels. Left Axis: palbociclib concentration (nM), red dotted curve, model estimated concentration. Horizontal black solid, green dashed, and red dotted lines: ANC thresholds of 1.5, 1.0, and 0.5 × 10^3^/µL, respectively; Figure S2_25: ANC vs Time Plots for patient #25. Right Axis: ANC (10^3^/µL), black curve, model estimated curve; black dots measured ANC levels. Left Axis: palbociclib concentration (nM), red dotted curve, model estimated concentration. Horizontal black solid, green dashed, and red dotted lines: ANC thresholds of 1.5, 1.0, and 0.5 × 10^3^/µL, respectively; Figure S3_1: PLT vs Time Plots for patient #1. Right Axis: PLT (10^9^/L), black curve, model estimated curve; black dots measured PLT levels. Left Axis: palbociclib concentration (nM), red dotted curve, model estimated concentration. Horizontal black solid, green dashed, and red dotted lines: PLT thresholds of 100, 50, and 25 × 10^9^/L, respectively; Figure S3_5: PLT vs Time Plots for patient #5. Right Axis: PLT (10^9^/L), black curve, model estimated curve; black dots measured PLT levels. Left Axis: palbociclib concentration (nM), red dotted curve, model estimated concentration. Horizontal black solid, green dashed, and red dotted lines: PLT thresholds of 100, 50, and 25 × 10^9^/L, respectively; Figure S3_14: PLT vs Time Plots for patient #14. Right Axis: PLT (10^9^/L), black curve, model estimated curve; black dots measured PLT levels. Left Axis: palbociclib concentration (nM), red dotted curve, model estimated concentration. Horizontal black solid, green dashed, and red dotted lines: PLT thresholds of 100, 50, and 25 × 10^9^/L, respectively; Figure S3_21: PLT vs Time Plots for patient #21. Right Axis: PLT (10^9^/L), black curve, model estimated curve; black dots measured PLT levels. Left Axis: palbociclib concentration (nM), red dotted curve, model estimated concentration. Horizontal black solid, green dashed, and red dotted lines: PLT thresholds of 100, 50, and 25 × 10^9^/L, respectively; Figure S3_22: PLT vs Time Plots for patient #22. Right Axis: PLT (10^9^/L), black curve, model estimated curve; black dots measured PLT levels. Left Axis: palbociclib concentration (nM), red dotted curve, model estimated concentration. Horizontal black solid, green dashed, and red dotted lines: PLT thresholds of 100, 50, and 25 × 10^9^/L, respectively; Figure S3_25: PLT vs Time Plots for patient #25. Right Axis: PLT (10^9^/L), black curve, model estimated curve; black dots measured PLT levels. Left Axis: palbociclib concentration (nM), red dotted curve, model estimated concentration. Horizontal black solid, green dashed, and red dotted lines: PLT thresholds of 100, 50, and 25 × 10^9^/L, respectively; Figure S4: Simulated ANC vs time given palbociclib dosages of 50, 75, and 95 mg/m^2^/day for 21 days for Stratum 1 and 2. Horizontal black solid, green dashed, and red dotted lines: ANC thresholds of 1.5, 1.0, and 0.5 × 10^3^/µL, respectively. Black curve: median ANC; Blue shaded region: 25–75th percentiles; Grey shaded region: 5–95th percentiles; Figure S5: Simulated PLT vs time given palbociclib dosages of 50, 75, and 95 mg/m^2^/day for 21 days for Stratum 1 and 2. Horizontal black solid, green dashed, and red dotted lines: PLT thresholds of 100, 50, and 25 × 10^9^/L, respectively. Black curve: median PLT; Blue shaded region: 25–75th percentiles; Grey shaded region: 5–95th percentiles.

## References

[1] Ostrom Q.T., Patil N., Cioffi G., Waite K., Kruchko C., Barnholtz-Sloan J.S. (2020). CBTRUS Statistical Report: Primary Brain and Other Central Nervous System Tumors Diagnosed in the United States in 2013–2017. Neuro-oncology.

[2] Plant-Fox A.S., O’Halloran K., Goldman S. (2021). Pediatric brain tumors: The era of molecular diagnostics, targeted and immune-based therapeutics, and a focus on long term neurologic sequelae. Curr. Probl. Cancer.

[3] Weiser A., Sanchez Bergman A., Machaalani C., Bennett J., Roth P., Reimann R.R., Nazarian J., Guerreiro Stucklin A.S. (2023). Bridging the age gap: A review of molecularly informed treatments for glioma in adolescents and young adults. Front. Oncol..

[4] Chen F., Chandrashekar D.S., Scheurer M.E., Varambally S., Creighton C.J. (2022). Global molecular alterations involving recurrence or progression of pediatric brain tumors. Neoplasia.

[5] Gajjar A., Mahajan A., Abdelbaki M., Anderson C., Antony R., Bale T., Bindra R., Bowers D.C., Cohen K., Cole B. (2022). Pediatric Central Nervous System Cancers, Version 2.2023, NCCN Clinical Practice Guidelines in Oncology. J. Natl. Compr. Cancer Netw..

[6] Miklja Z., Pasternak A., Stallard S., Nicolaides T., Kline-Nunnally C., Cole B., Beroukhim R., Bandopadhayay P., Chi S., Ramkissoon S.H. (2019). Molecular profiling and targeted therapy in pediatric gliomas: Review and consensus recommendations. Neuro-oncology.

[7] Paugh B.S., Zhu X., Qu C., Endersby R., Diaz A.K., Zhang J., Bax D.A., Carvalho D., Reis R.M., Onar-Thomas A. (2013). Novel oncogenic PDGFRA mutations in pediatric high-grade gliomas. Cancer Res..

[8] Van Mater D., Gururangan S., Becher O., Campagne O., Leary S., Phillips J.J., Huang J., Lin T., Poussaint T.Y., Goldman S. (2021). A phase I trial of the CDK 4/6 inhibitor palbociclib in pediatric patients with progressive brain tumors: A Pediatric Brain Tumor Consortium study (PBTC-042). Pediatr. Blood Cancer.

[9] Glade Bender J.L., Lee A., Reid J.M., Baruchel S., Roberts T., Voss S.D., Wu B., Ahern C.H., Ingle A.M., Harris P. (2013). Phase I pharmacokinetic and pharmacodynamic study of pazopanib in children with soft tissue sarcoma and other refractory solid tumors: A children’s oncology group phase I consortium report. J. Clin. Oncol..

[10] Spring L.M., Zangardi M.L., Moy B., Bardia A. (2017). Clinical Management of Potential Toxicities and Drug Interactions Related to Cyclin-Dependent Kinase 4/6 Inhibitors in Breast Cancer: Practical Considerations and Recommendations. Oncologist.

[11] Hu W., Sung T., Jessen B.A., Thibault S., Finkelstein M.B., Khan N.K., Sacaan A.I. (2016). Mechanistic Investigation of Bone Marrow Suppression Associated with Palbociclib and its Differentiation from Cytotoxic Chemotherapies. Clin. Cancer Res..

[12] Finn R.S., Martin M., Rugo H.S., Jones S., Im S.A., Gelmon K., Harbeck N., Lipatov O.N., Walshe J.M., Moulder S. (2016). Palbociclib and Letrozole in Advanced Breast Cancer. N. Engl. J. Med..

[13] Turner N.C., Ro J., Andre F., Loi S., Verma S., Iwata H., Harbeck N., Loibl S., Huang Bartlett C., Zhang K. (2015). Palbociclib in Hormone-Receptor-Positive Advanced Breast Cancer. N. Engl. J. Med..

[14] Sun W., Klamerus K.J., Yuhas L.M., Pawlak S., Plotka A., O’Gorman M., Kirkovsky L., Kosa M., Wang D. (2017). Impact of Acid-Reducing Agents on the Pharmacokinetics of Palbociclib, a Weak Base with pH-Dependent Solubility, with Different Food Intake Conditions. Clin. Pharmacol. Drug Dev..

[15] Royer B., Kaderbhaï C., Fumet J.D., Hennequin A., Desmoulins I., Ladoire S., Ayati S., Mayeur D., Ilie S., Schmitt A. (2021). Population Pharmacokinetics of Palbociclib in a Real-World Situation. Pharmaceuticals.

[16] Marouille A.L., Petit E., Kaderbhaï C., Desmoulins I., Hennequin A., Mayeur D., Fumet J.D., Ladoire S., Tharin Z., Ayati S. (2021). Pharmacokinetic/Pharmacodynamic Model of Neutropenia in Real-Life Palbociclib-Treated Patients. Pharmaceutics.

[17] Beal S.L. (2001). Ways to fit a PK model with some data below the quantification limit. J. Pharmacokinet. Pharmacodyn..

[18] Millisor V.E., Roberts J.K., Sun Y., Tang L., Daryani V.M., Gregornik D., Cross S.J., Ward D., Pauley J.L., Molinelli A. (2017). Derivation of new equations to estimate glomerular filtration rate in pediatric oncology patients. Pediatr. Nephrol..

[19] Reddick S.J., Campagne O., Huang J., Onar-Thomas A., Broniscer A., Gajjar A., Stewart C.F. (2019). Pharmacokinetics and safety of erlotinib and its metabolite OSI-420 in infants and children with primary brain tumors. Cancer Chemother. Pharmacol..

[20] Campagne O., Zhong B., Nair S., Lin T., Huang J., Onar-Thomas A., Robinson G., Gajjar A., Stewart C.F. (2020). Exposure-Toxicity Association of Cyclophosphamide and Its Metabolites in Infants and Young Children with Primary Brain Tumors: Implications for Dosing. Clin. Cancer Res..

[21] Panetta J.C., Schaiquevich P., Santana V.M., Stewart C.F. (2008). Using pharmacokinetic and pharmacodynamic modeling and simulation to evaluate importance of schedule in topotecan therapy for pediatric neuroblastoma. Clin. Cancer Res..

[22] Raetz E.A., Teachey D.T., Minard C., Liu X., Norris R.E., Denic K.Z., Reid J., Evensen N.A., Gore L., Fox E. (2023). Palbociclib in combination with chemotherapy in pediatric and young adult patients with relapsed/refractory acute lymphoblastic leukemia and lymphoma: A Children’s Oncology Group study (AINV18P1). Pediatr. Blood Cancer.

[23] Bandiera C., Cardoso E., Locatelli I., Digklia A., Zaman K., Diciolla A., Cristina V., Stravodimou A., Veronica A.L., Dolcan A. (2021). Optimizing Oral Targeted Anticancer Therapies Study for Patients With Solid Cancer: Protocol for a Randomized Controlled Medication Adherence Program Along With Systematic Collection and Modeling of Pharmacokinetic and Pharmacodynamic Data. JMIR Res. Protoc..

[24] Schwartz G.K., LoRusso P.M., Dickson M.A., Randolph S.S., Shaik M.N., Wilner K.D., Courtney R., O’Dwyer P.J. (2011). Phase I study of PD 0332991, a cyclin-dependent kinase inhibitor, administered in 3-week cycles (Schedule 2/1). Br. J. Cancer.

[25] Flaherty K.T., Lorusso P.M., Demichele A., Abramson V.G., Courtney R., Randolph S.S., Shaik M.N., Wilner K.D., O’Dwyer P.J., Schwartz G.K. (2012). Phase I, dose-escalation trial of the oral cyclin-dependent kinase 4/6 inhibitor PD 0332991, administered using a 21-day schedule in patients with advanced cancer. Clin. Cancer Res..

[26] Friberg L.E., Henningsson A., Maas H., Nguyen L., Karlsson M.O. (2002). Model of chemotherapy-induced myelosuppression with parameter consistency across drugs. J. Clin. Oncol..

[27] Sun W., O’Dwyer P.J., Finn R.S., Ruiz-Garcia A., Shapiro G.I., Schwartz G.K., DeMichele A., Wang D. (2017). Characterization of Neutropenia in Advanced Cancer Patients Following Palbociclib Treatment Using a Population Pharmacokinetic-Pharmacodynamic Modeling and Simulation Approach. J. Clin. Pharmacol..

[28] Jian W., Xue J., Yao Q., Chen R., Yao Y., Wang M., Zhou T. (2022). Starting dose selection of palbociclib in Chinese patients with breast cancer based on population kinetic-pharmacodynamic model of neutropenia. Cancer Chemother. Pharmacol..

[29] Lu Y., Yang S., Ho Y.Y., Ji Y. (2021). Ribociclib Population Pharmacokinetics and Pharmacokinetic/Pharmacodynamic Analysis of Neutrophils in Cancer Patients. J Clin. Pharmacol..

[30] Li C., Hart L., Owonikoko T.K., Aljumaily R., Rocha Lima C.M., Conkling P.R., Webb R.T., Jotte R.M., Schuster S., Edenfield W.J. (2021). Trilaciclib dose selection: An integrated pharmacokinetic and pharmacodynamic analysis of preclinical data and Phase Ib/IIa studies in patients with extensive-stage small cell lung cancer. Cancer Chemother. Pharmacol..

[31] Panetta J.C., Kirstein M.N., Gajjar A.J., Nair G., Fouladi M., Stewart C.F. (2003). A mechanistic mathematical model of temozolomide myelosuppression in children with high-grade gliomas. Math. Biosci..

