# Supplementary material for: Population Pharmacokinetic and Pharmacodynamic Study of Palbociclib in Children and Young Adults with Recurrent, Progressive, or Refractory Brain Tumors
Source: Pharmaceutics. 2024 Nov 28;16(12):1528. doi: 10.3390/pharmaceutics16121528 (PMC11676693; doi:10.3390/pharmaceutics16121528)
Supplement: Supplementary file 1 [file pharmaceutics-16-01528-s001.zip › pharmaceutics-3300843-supplementary.pdf]

Population Pharmacokinetic and Pharmacodynamic Study of Palbociclib in Children and Young Adults with Recurrent, Progressive, or Refractory Brain Tumors

SUPPLEMENTARY MATERIAL

Corresponding author: Clinton F. Stewart, Department of Pharmacy and Pharmaceutical Sciences, St. Jude Children's Research Hospital, 262 Danny Thomas Place, Memphis, TN 38105, USA, clinton.stewart@stjude.org; Tel.: +1-901-595-3665; Fax: +1-901+525-6869

CONTENTS:

Page	Label	Description
3	Table S1	Comparison of the base pharmacokinetic model's objective function value (OFV) with models including single covariates on apparent clearance (Cl/F).
4	Figure S1	Depicted below is the pharmacodynamic model scheme describing the ANC and platelet dynamics. The model for ANC consists of a bone marrow compartment with proliferating neutrophils, three transient compartments that represent the maturation process of the neutrophils from blasts to circulating neutrophils, and a circulating neutrophil compartment. The model included the negative feedback effects of endogenous G-CSF, which affected the growth rate of proliferating cells in a manner inversely proportional to the concentration of circulating neutrophils. We assumed that palbociclib only affected the proliferating neutrophils in the bone marrow compartment. We used a similar structural model for platelet dynamics.
4	Figure S2_1	ANC vs Time Plots for patient #1. Right Axis: ANC (10^3/μL), black curve, model estimated curve; black dots measured ANC levels. Left Axis: palbociclib concentration (nM), red dotted curve, model estimated concentration. Horizontal black solid, green dashed, and red dotted lines: ANC thresholds of 1.5, 1.0, and 0.5 $\times 10^3$/μL, respectively.
5	Figure S2_5	ANC vs Time Plots for patient #5. Right Axis: ANC (10^3/μL), black curve, model estimated curve; black dots measured ANC levels. Left Axis: palbociclib concentration (nM), red dotted curve, model estimated concentration. Horizontal black solid, green dashed, and red dotted lines: ANC thresholds of 1.5, 1.0, and 0.5 $\times 10^3$/μL, respectively.
6	Figure S2_14	ANC vs Time Plots for patient #14. Right Axis: ANC (10^3/μL), black curve, model estimated curve; black dots measured ANC levels. Left Axis: palbociclib concentration (nM), red dotted curve, model estimated concentration. Horizontal black solid, green dashed, and red dotted lines: ANC thresholds of 1.5, 1.0, and 0.5 $\times 10^3$/μL, respectively.
7	Figure S2_21	ANC vs Time Plots for patient #21. Right Axis: ANC (10^3/μL), black curve, model estimated curve; black dots measured ANC levels. Left Axis: palbociclib concentration (nM), red dotted curve, model estimated concentration. Horizontal black solid, green dashed, and red dotted lines: ANC thresholds of 1.5, 1.0, and 0.5 $\times 10^3$/μL, respectively.

8	Figure S2_22	ANC vs Time Plots for patient #22. Right Axis: ANC (10^3/μL), black curve, model estimated curve; black dots measured ANC levels. Left Axis: palbociclib concentration (nM), red dotted curve, model estimated concentration. Horizontal black solid, green dashed, and red dotted lines: ANC thresholds of 1.5, 1.0, and 0.5 $\times 10^3$/μL, respectively.
9	Figure S2_25	ANC vs Time Plots for patient #25. Right Axis: ANC (10^3/μL), black curve, model estimated curve; black dots measured ANC levels. Left Axis: palbociclib concentration (nM), red dotted curve, model estimated concentration. Horizontal black solid, green dashed, and red dotted lines: ANC thresholds of 1.5, 1.0, and 0.5 $\times 10^3$/μL, respectively.
10	Figure S3_1	PLT vs Time Plots for patient #1. Right Axis: PLT (10^9/L), black curve, model estimated curve; black dots measured PLT levels. Left Axis: palbociclib concentration (nM), red dotted curve, model estimated concentration. Horizontal black solid, green dashed, and red dotted lines: PLT thresholds of 100, 50, and 25 $\times 10^9$/L, respectively.
11	Figure S3_5	PLT vs Time Plots for patient #5. Right Axis: PLT (10^9/L), black curve, model estimated curve; black dots measured PLT levels. Left Axis: palbociclib concentration (nM), red dotted curve, model estimated concentration. Horizontal black solid, green dashed, and red dotted lines: PLT thresholds of 100, 50, and 25 $\times 10^9$/L, respectively.
12	Figure S3_14	PLT vs Time Plots for patient #14. Right Axis: PLT (10^9/L), black curve, model estimated curve; black dots measured PLT levels. Left Axis: palbociclib concentration (nM), red dotted curve, model estimated concentration. Horizontal black solid, green dashed, and red dotted lines: PLT thresholds of 100, 50, and 25 $\times 10^9$/L, respectively.
13	Figure S3_21	PLT vs Time Plots for patient #21. Right Axis: PLT (10^9/L), black curve, model estimated curve; black dots measured PLT levels. Left Axis: palbociclib concentration (nM), red dotted curve, model estimated concentration. Horizontal black solid, green dashed, and red dotted lines: PLT thresholds of 100, 50, and 25 $\times 10^9$/L, respectively.
14	Figure S3_22	PLT vs Time Plots for patient #22. Right Axis: PLT (10^9/L), black curve, model estimated curve; black dots measured PLT levels. Left Axis: palbociclib concentration (nM), red dotted curve, model estimated concentration. Horizontal black solid, green dashed, and red dotted lines: PLT thresholds of 100, 50, and 25 $\times 10^9$/L, respectively.
15	Figure S3_25	PLT vs Time Plots for patient #25. Right Axis: PLT (10^9/L), black curve, model estimated curve; black dots measured PLT levels. Left Axis: palbociclib concentration (nM), red dotted curve, model estimated concentration. Horizontal black solid, green dashed, and red dotted lines: PLT thresholds of 100, 50, and 25 $\times 10^9$/L, respectively.
16 to 21	Figure S4	Simulated ANC vs time given palbociclib dosages of 50, 75, and 95 mg/m^2/day for 21 days for Stratum 1 and 2. Horizontal black solid, green dashed, and red dotted lines: ANC thresholds of 1.5, 1.0, and 0.5 $\times 10^3$/μL, respectively. Black curve: median ANC; Blue shaded region: 25th-75th percentiles; Grey shaded region: 5th-95th percentiles.
22 to 27	Figure S5	Simulated PLT vs time given palbociclib dosages of 50, 75, and 95 mg/m^2/day for 21 days for Stratum 1 and 2. Horizontal black solid, green dashed, and red dotted lines: PLT thresholds of 100, 50, and 25 $\times 10^9$/L, respectively. Black curve: median PLT; Blue shaded region: 25th-75th percentiles; Grey shaded region: 5th-95th percentiles.

Table S1: Comparison of the base pharmacokinetic model's objective function value (OFV) with models including single covariates on apparent clearance (Cl/F).

PK model*			2* Log Likelihood value (OFV)	Decrease in OFV compared to the base model.	P-value
Base model			3198.77		
Aspartate aminotransferase			3191.39	-7.38	6.6E-03
**Concomitant Drugs**	**# Patients**	**# Studies**			
Ondansetron	2	4	3194.98	-3.79	5.2E-02
Levothyroxine	5	6	3195.24	-3.53	6.0E-02
Granisetron	5	8	3195.82	-2.95	8.6E-02
Dexamethasone	8	13	3196.19	-2.58	1.1E-01
Lacosamide	3	5	3197.04	-1.73	1.9E-01
Levetiracetam	12	23	3197.45	-1.32	2.5E-01
Gabapentin	3	6	3197.69	-1.08	3.0E-01
Trimethoprim/Sulfamethoxazole	3	4	3197.89	-0.88	3.5E-01
Famotidine	1	2	3198.19	-0.58	4.5E-01
Lorazepam	4	5	3198.45	-0.32	5.7E-01
Ranitidine	3	5	3198.76	-0.01	9.2E-01

*Covariates added to the Base model

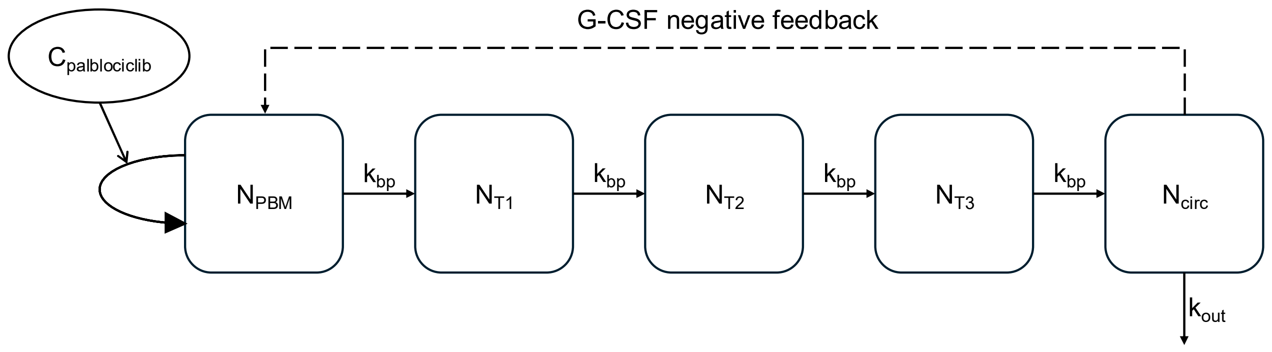

Figure S1. Depicted below is the pharmacodynamic model scheme describing the ANC and platelet dynamics. The model for ANC consists of a bone marrow compartment with proliferating neutrophils, three transient compartments that represent the maturation process of the neutrophils from blasts to circulating neutrophils, and a circulating neutrophil compartment. The model included the negative feedback effects of endogenous G-CSF, which affected the growth rate of proliferating cells in a manner inversely proportional to the concentration of circulating neutrophils. We assumed that palbociclib only affected the proliferating neutrophils in the bone marrow compartment. We used a similar structural model for platelet dynamics.

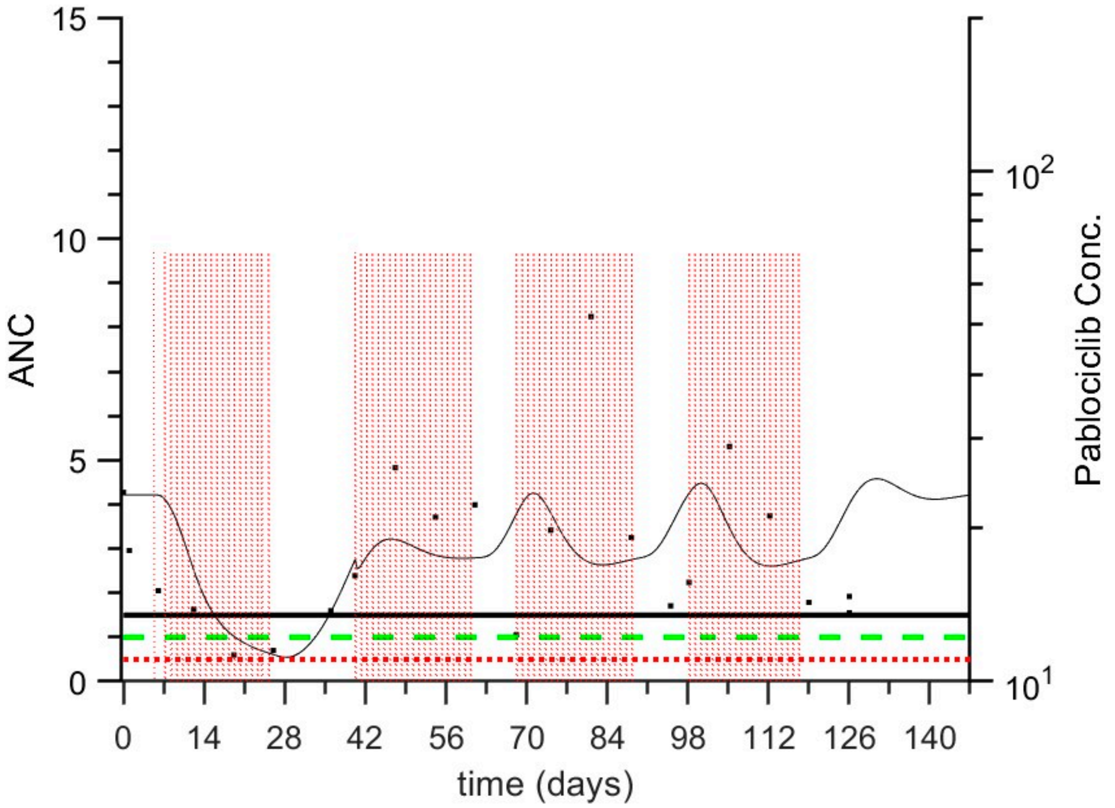

Figure S2_1. ANC vs Time Plots for patient #1. Right Axis: ANC (10^3/μL), black curve, model estimated curve; black dots measured ANC levels. Left Axis: palbociclib concentration (nM), red dotted curve, model estimated concentration. Horizontal black solid, green dashed, and red dotted lines: ANC thresholds of 1.5, 1.0, and 0.5 $\times 10^3$/μL, respectively.

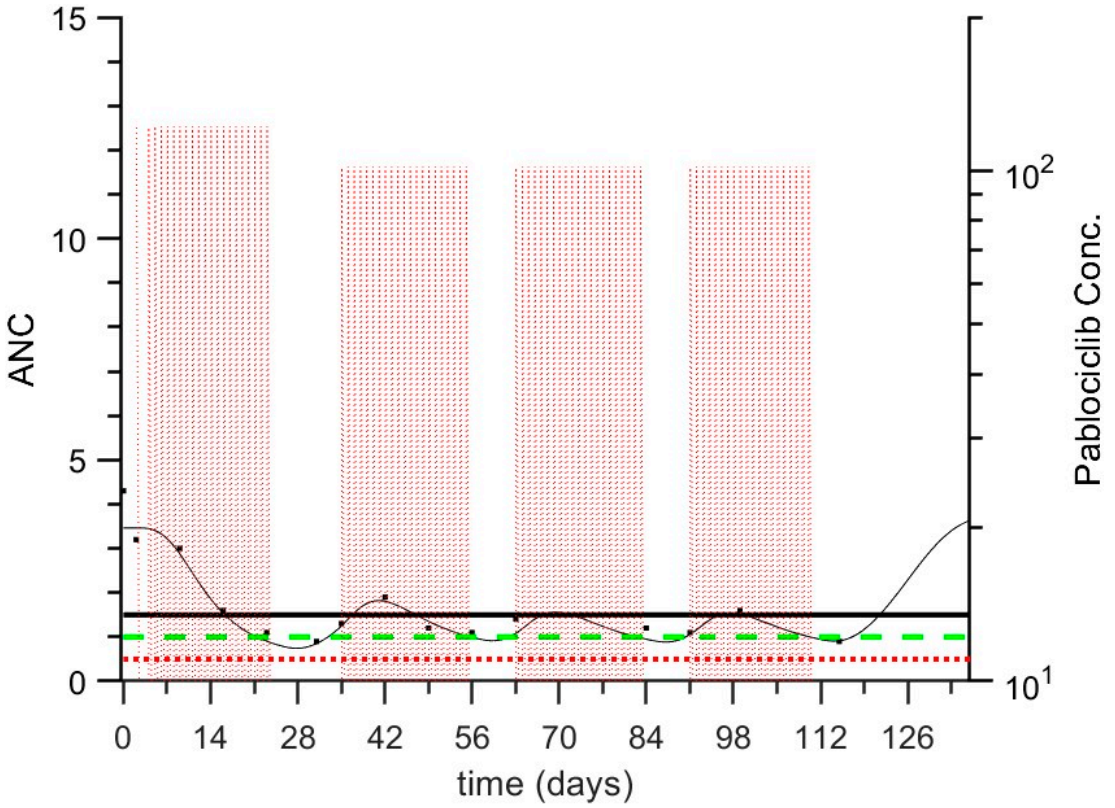

Figure S2_5. ANC vs Time Plots for patient #5. Right Axis: ANC (10^3/μL), black curve, model estimated curve; black dots measured ANC levels. Left Axis: palbociclib concentration (nM), red dotted curve, model estimated concentration. Horizontal black solid, green dashed, and red dotted lines: ANC thresholds of 1.5, 1.0, and 0.5 $\times 10^3$/μL, respectively.

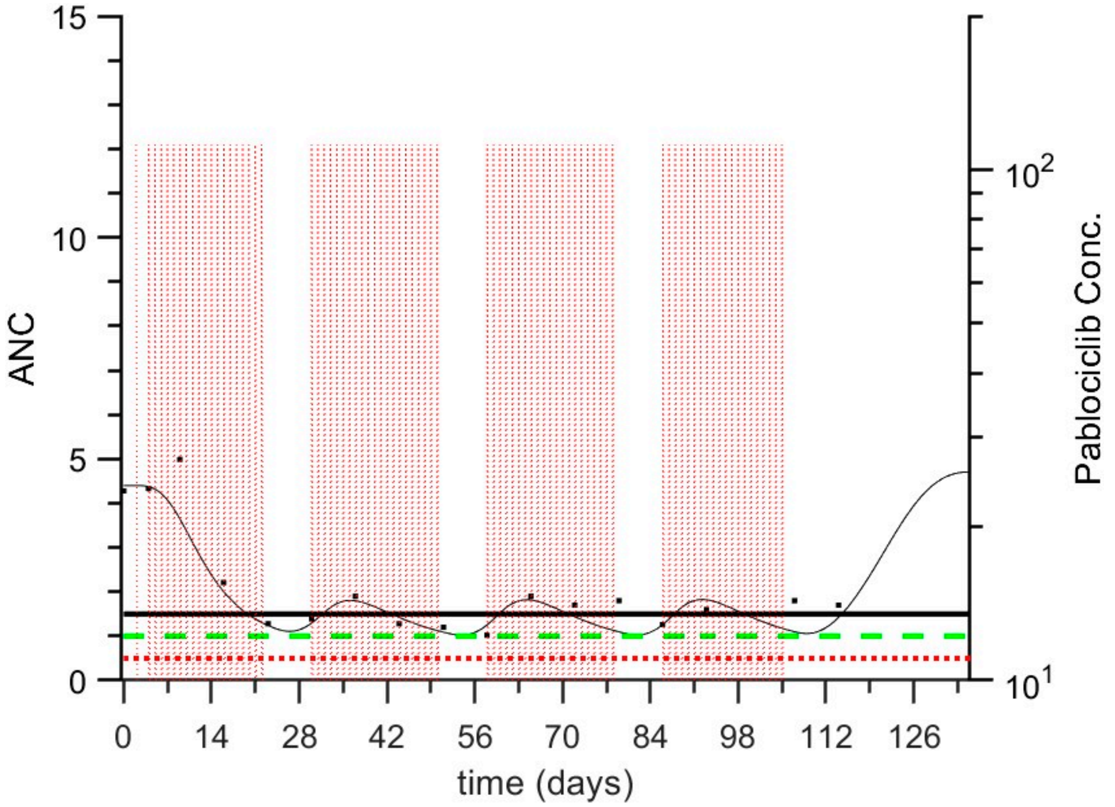

Figure S2_14. ANC vs Time Plots for patient #14. Right Axis: ANC (10^3/µL), black curve, model estimated curve; black dots measured ANC levels. Left Axis: palbociclib concentration (nM), red dotted curve, model estimated concentration. Horizontal black solid, green dashed, and red dotted lines: ANC thresholds of 1.5, 1.0, and 0.5 $\times 10^3$/µL, respectively.

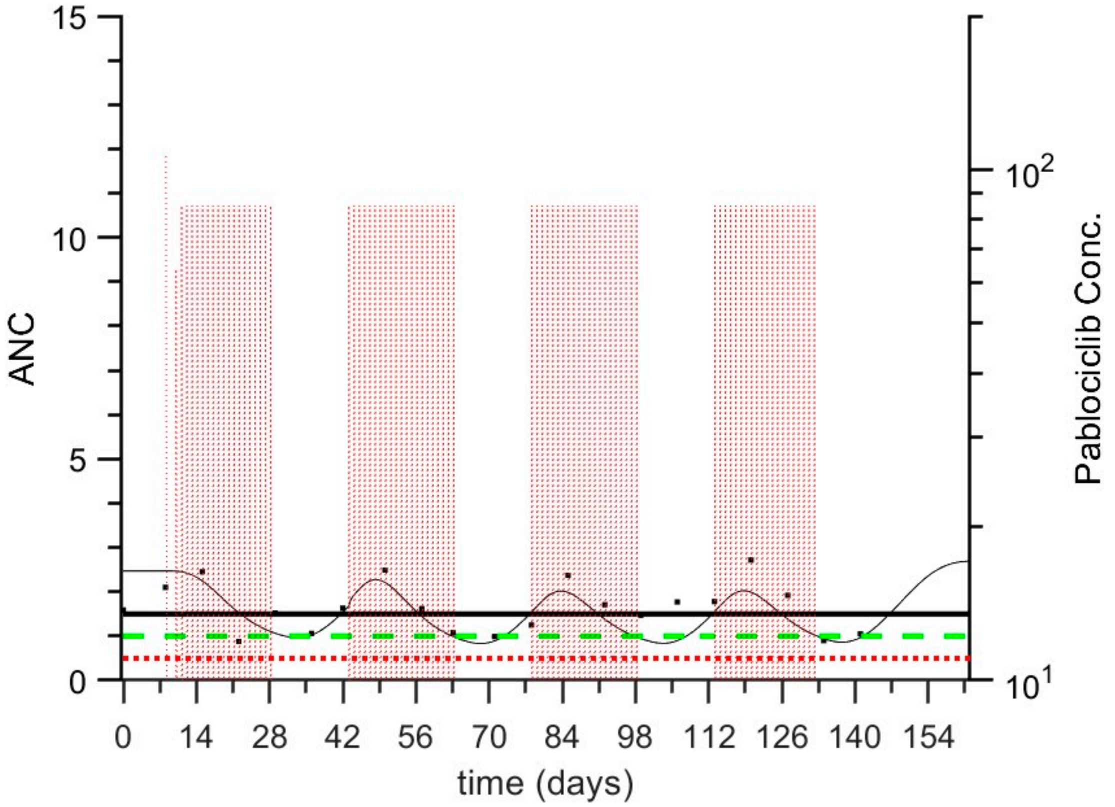

Figure S2_21. ANC vs Time Plots for patient #21. Right Axis: ANC (10^3/µL), black curve, model estimated curve; black dots measured ANC levels. Left Axis: palbociclib concentration (nM), red dotted curve, model estimated concentration. Horizontal black solid, green dashed, and red dotted lines: ANC thresholds of 1.5, 1.0, and 0.5 $\times 10^3$/µL, respectively.

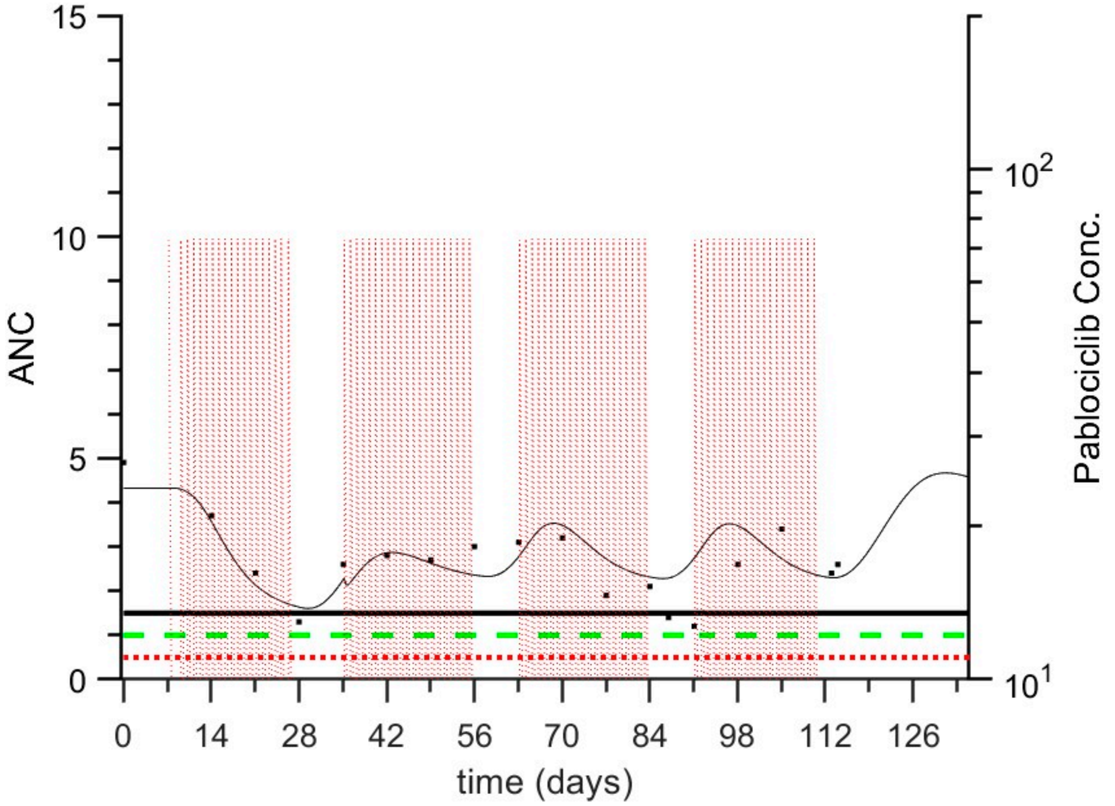

Figure S2_22. ANC vs Time Plots for patient #22. Right Axis: ANC (10^3/μL), black curve, model estimated curve; black dots measured ANC levels. Left Axis: palbociclib concentration (nM), red dotted curve, model estimated concentration. Horizontal black solid, green dashed, and red dotted lines: ANC thresholds of 1.5, 1.0, and 0.5 $\times 10^3$/μL, respectively.

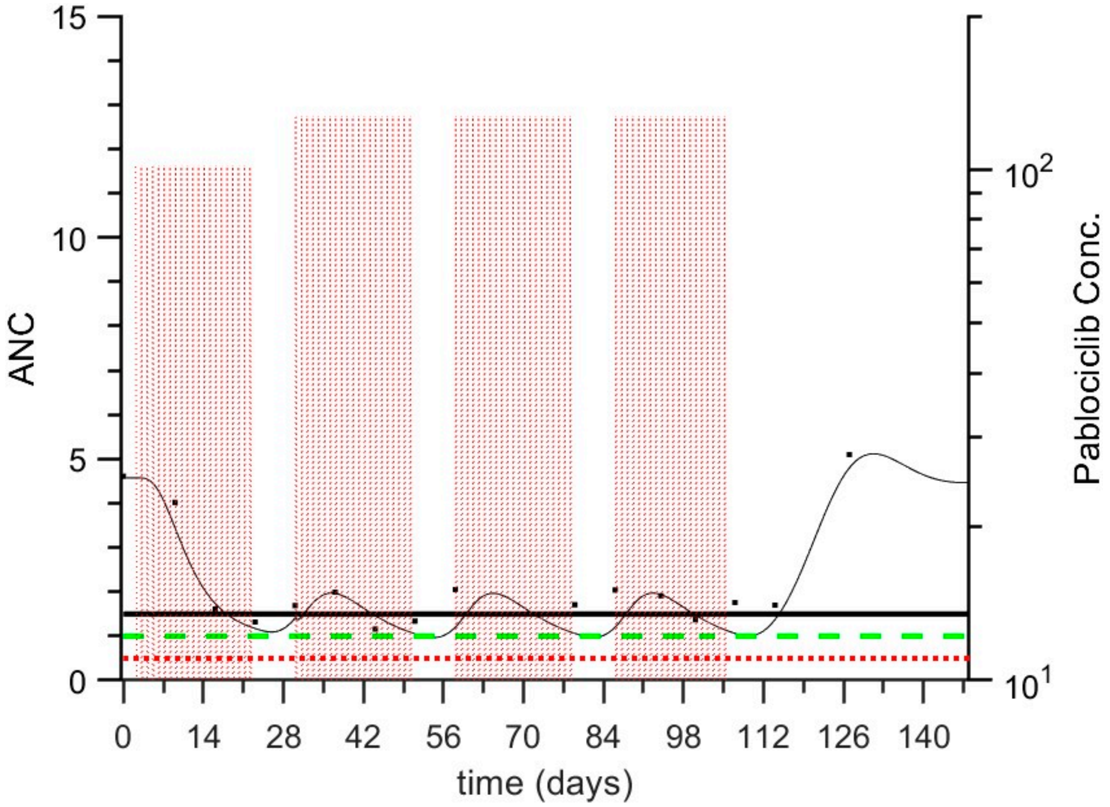

Figure S2_25. ANC vs Time Plots for patient #25. Right Axis: ANC (10^3/μL), black curve, model estimated curve; black dots measured ANC levels. Left Axis: palbociclib concentration (nM), red dotted curve, model estimated concentration. Horizontal black solid, green dashed, and red dotted lines: ANC thresholds of 1.5, 1.0, and 0.5 $\times 10^3$/μL, respectively.

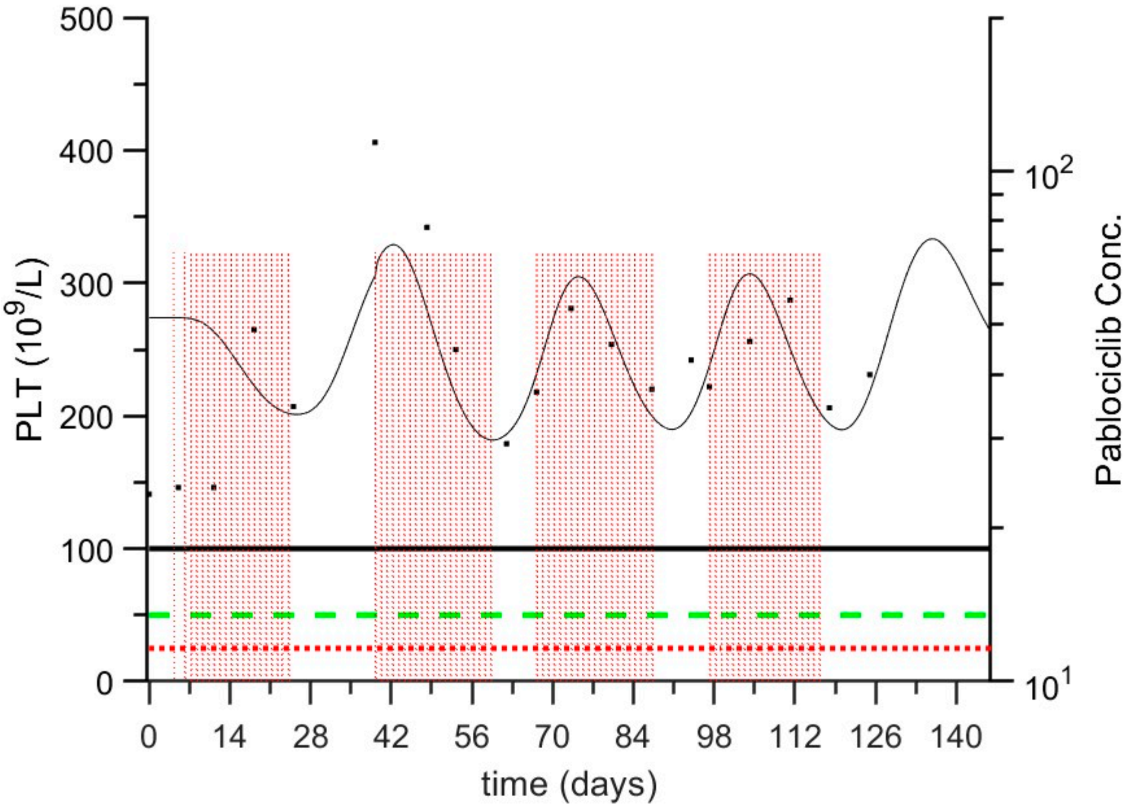

Figure S3_1. PLT vs Time Plots for patient #1. Right Axis: PLT (10^9/L), black curve, model estimated curve; black dots measured PLT levels. Left Axis: palbociclib concentration (nM), red dotted curve, model estimated concentration. Horizontal black, black solid, green dashed, and red dotted lines: PLT thresholds of 100, 75, 50, and 25 $\times 10^9$/L, respectively.

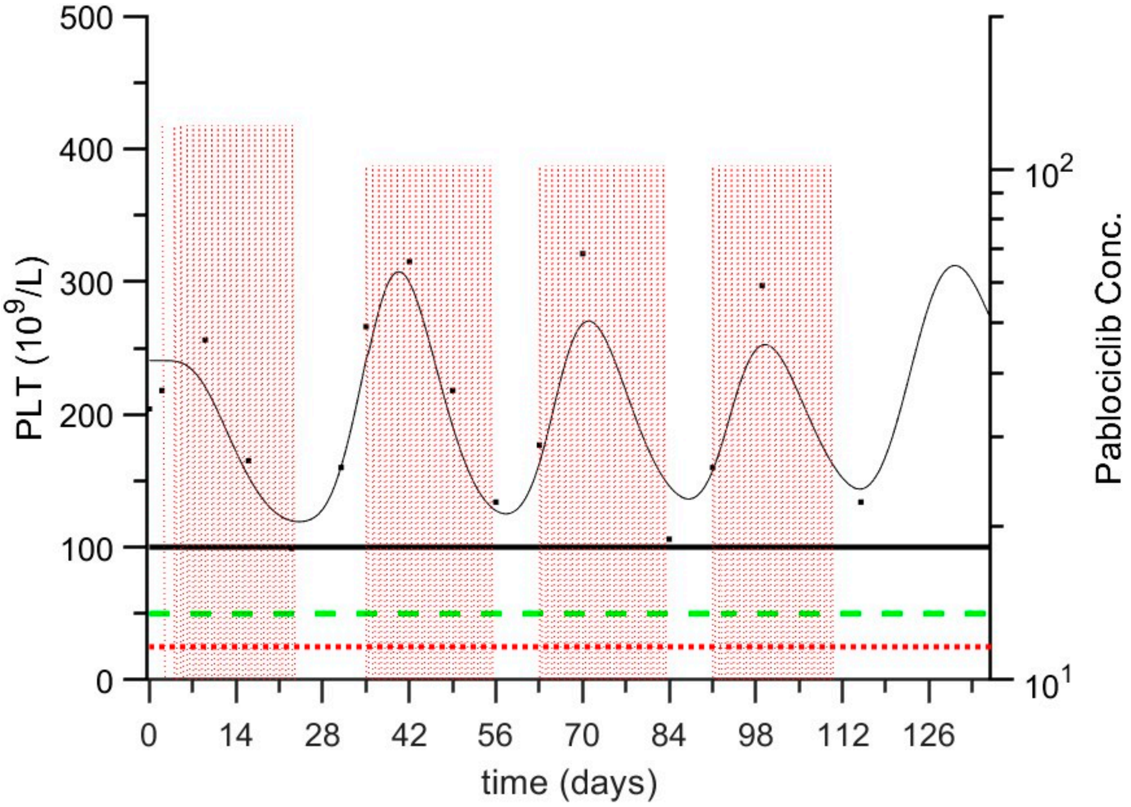

Figure S3_5. PLT vs Time Plots for patient #5. Right Axis: PLT (10^9/L), black curve, model estimated curve; black dots measured PLT levels. Left Axis: palbociclib concentration (nM), red dotted curve, model estimated concentration. Horizontal black, black solid, green dashed, and red dotted lines: PLT thresholds of 100, 75, 50, and 25 $\times 10^9$/L, respectively.

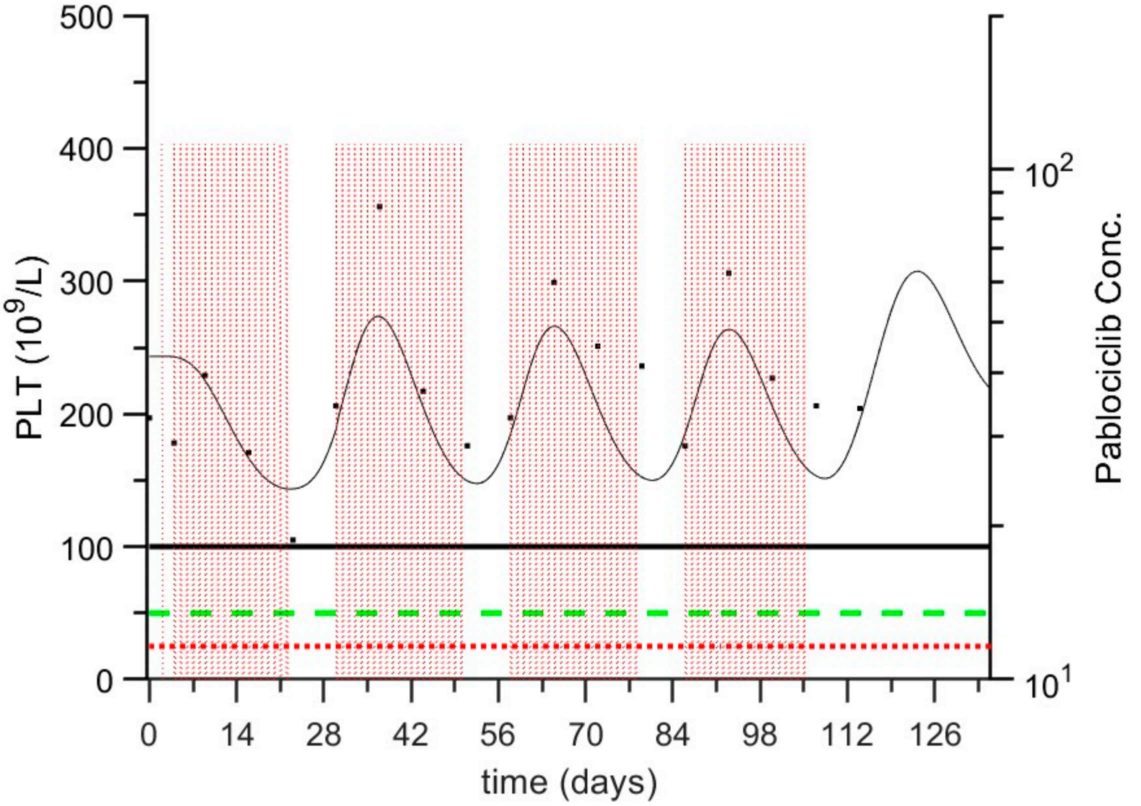

Figure S3_14. PLT vs Time Plots for patient #14. Right Axis: PLT (10^9/L), black curve, model estimated curve; black dots measured PLT levels. Left Axis: palbociclib concentration (nM), red dotted curve, model estimated concentration. Horizontal black, black solid, green dashed, and red dotted lines: PLT thresholds of 100, 75, 50, and 25 $\times 10^9$/L, respectively.

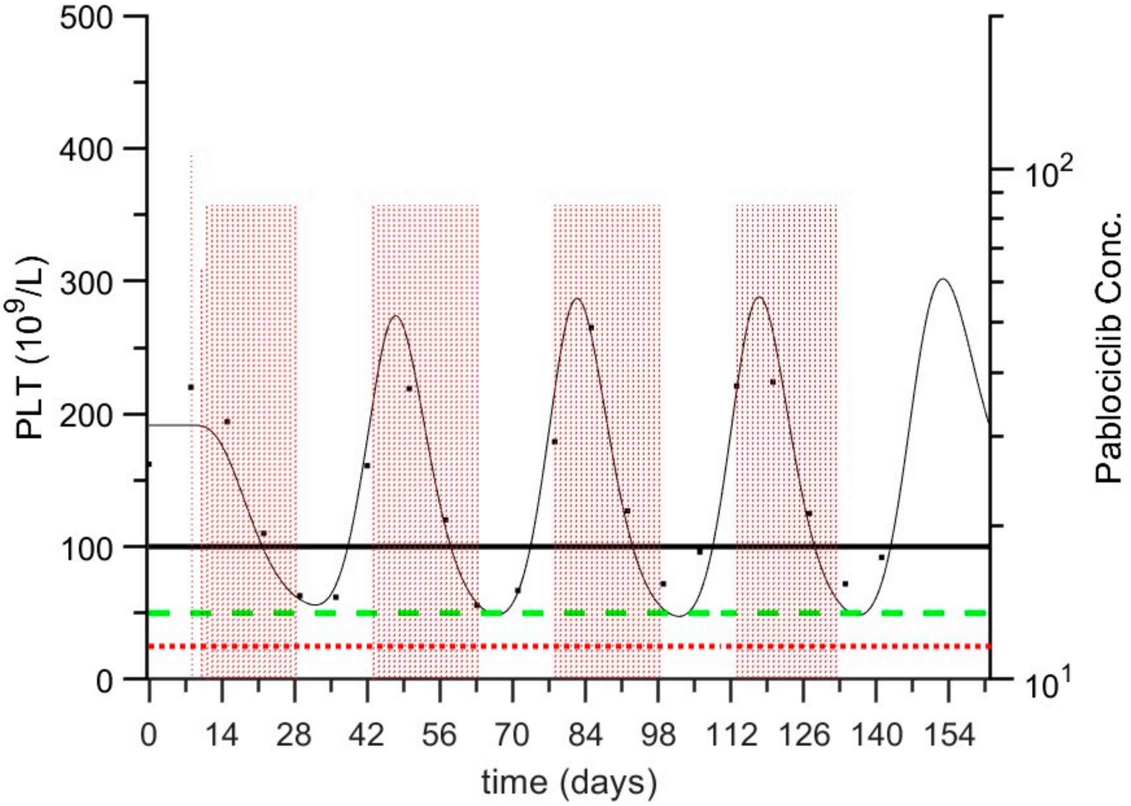

Figure S3_21. PLT vs Time Plots for patient #21. Right Axis: PLT (10^9/L), black curve, model estimated curve; black dots measured PLT levels. Left Axis: palbociclib concentration (nM), red dotted curve, model estimated concentration. Horizontal black, black solid, green dashed, and red dotted lines: PLT thresholds of 100, 75, 50, and 25 $\times 10^9$/L, respectively.

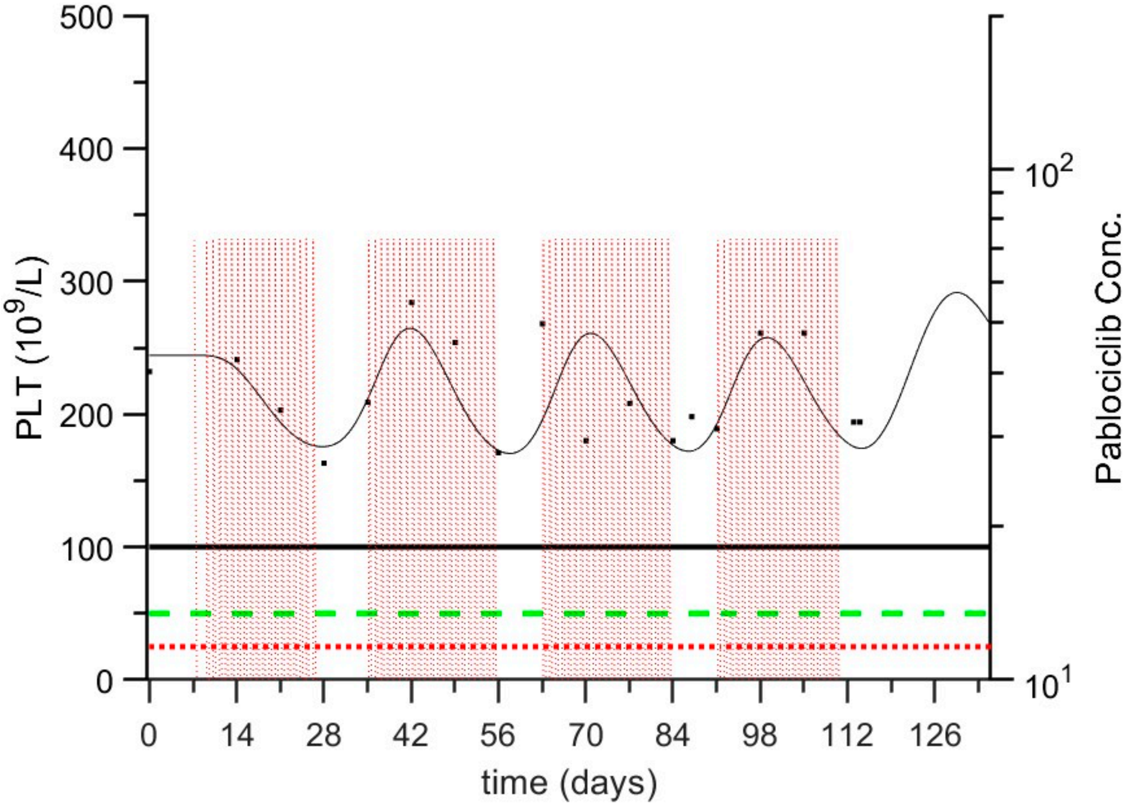

Figure S3_22. PLT vs Time Plots for patient #22. Right Axis: PLT (10^9/L), black curve, model estimated curve; black dots measured PLT levels. Left Axis: palbociclib concentration (nM), red dotted curve, model estimated concentration. Horizontal black, black solid, green dashed, and red dotted lines: PLT thresholds of 100, 75, 50, and 25 $\times 10^9$/L, respectively.

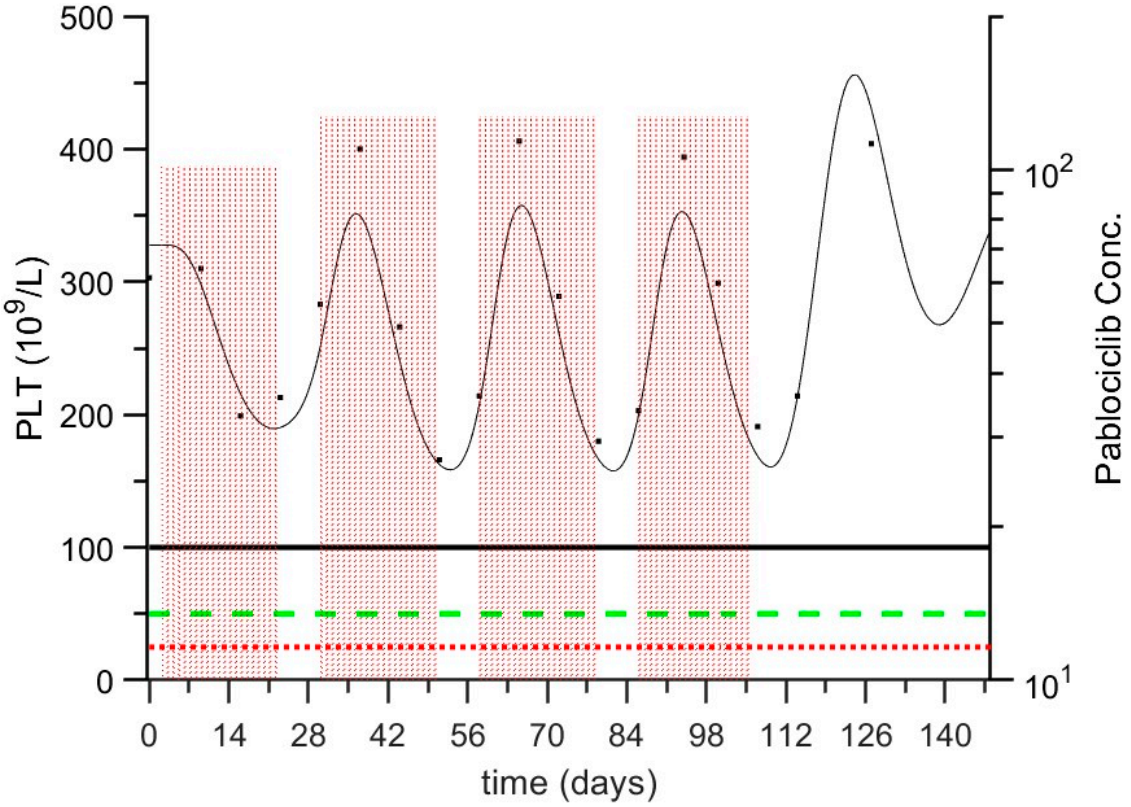

Figure S3_25. PLT vs Time Plots for patient #25. Right Axis: PLT (10^9/L), black curve, model estimated curve; black dots measured PLT levels. Left Axis: palbociclib concentration (nM), red dotted curve, model estimated concentration. Horizontal black, black solid, green dashed, and red dotted lines: PLT thresholds of 100, 75, 50, and 25 $\times 10^9$/L, respectively.

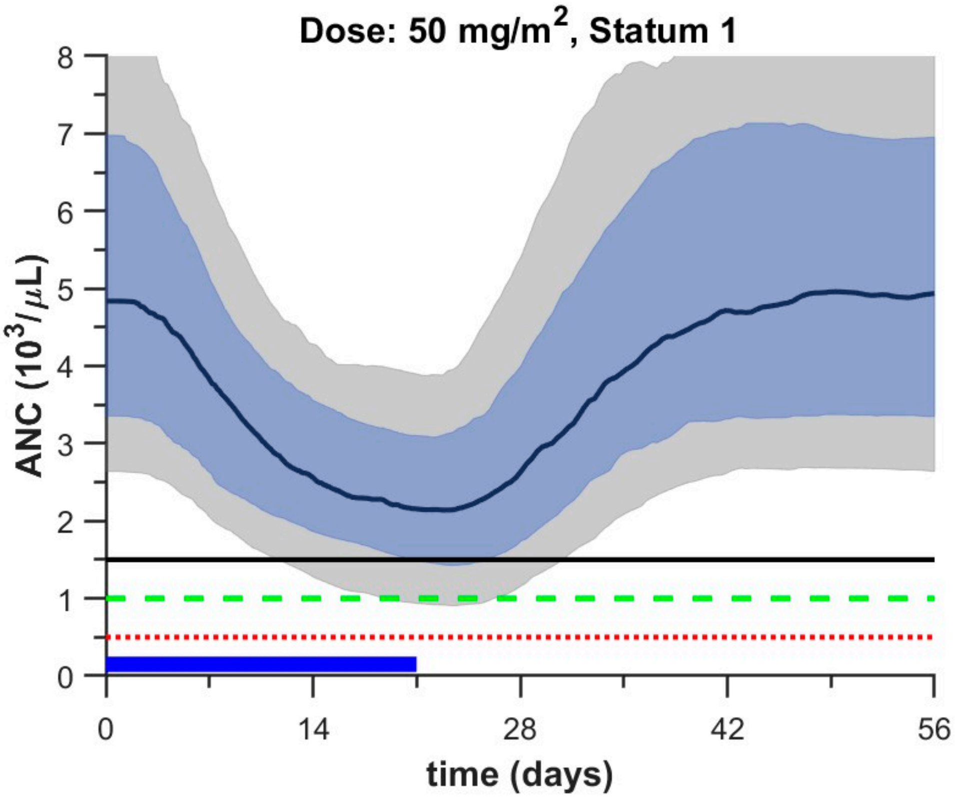

Figure S4. Simulated ANC vs time given a palbociclib dosage of 50 mg/m^2/day for 21 days for Stratum 1. Horizontal black solid, green dashed, and red dotted lines: ANC thresholds of 1.5, 1.0, and 0.5 $\times 10^3$/µL, respectively. Black curve: median ANC; Blue shaded region: 25th-75th percentiles; Grey shaded region: 5th-95th percentiles.

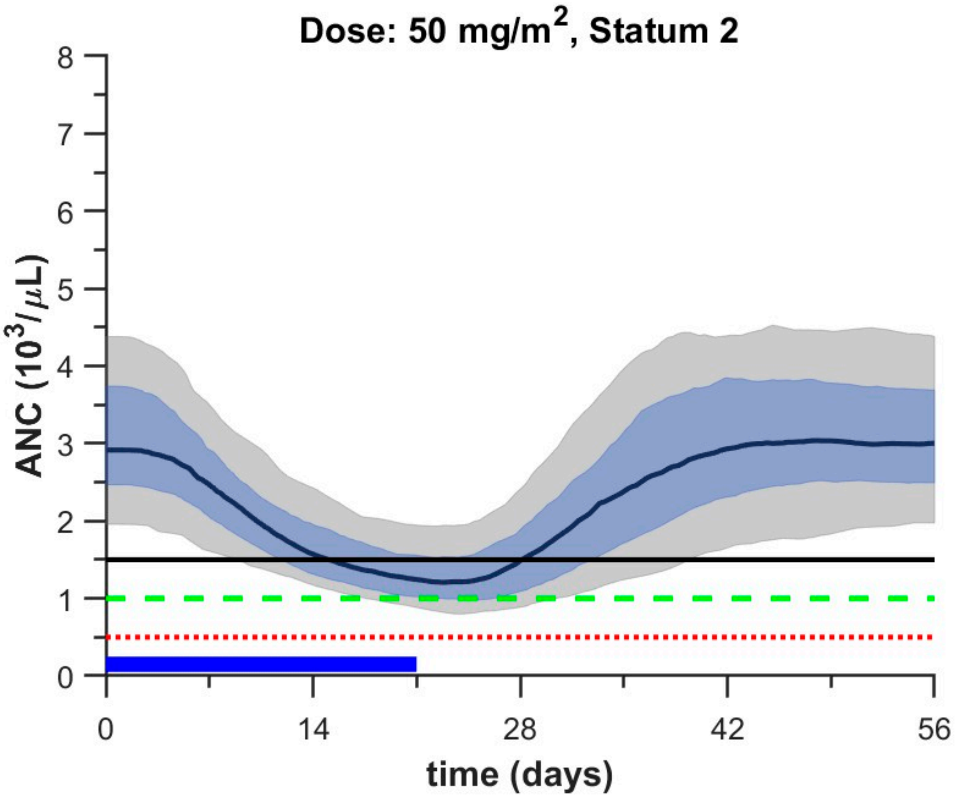

Figure S4. Simulated ANC vs time given a palbociclib dosage of 50 mg/m^2/day for 21 days for Stratum 2. Horizontal black solid, green dashed, and red dotted lines: ANC thresholds of 1.5, 1.0, and 0.5 $\times 10^3$/μL, respectively. Black curve: median ANC; Blue shaded region: 25th-75th percentiles; Grey shaded region: 5th-95th percentiles.

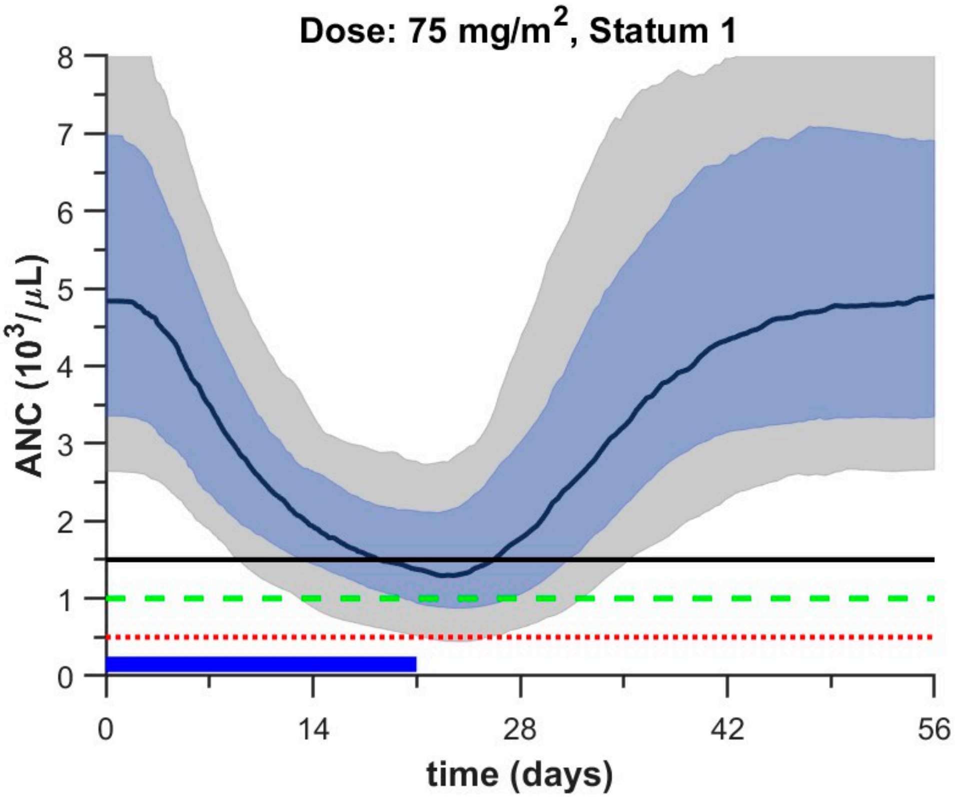

Figure S4. Simulated ANC vs time given a palbociclib dosage of 75 mg/m^2/day for 21 days for Stratum 1. Horizontal black solid, green dashed, and red dotted lines: ANC thresholds of 1.5, 1.0, and 0.5 $\times 10^3$/μL, respectively. Black curve: median ANC; Blue shaded region: 25th-75th percentiles; Grey shaded region: 5th-95th percentiles.

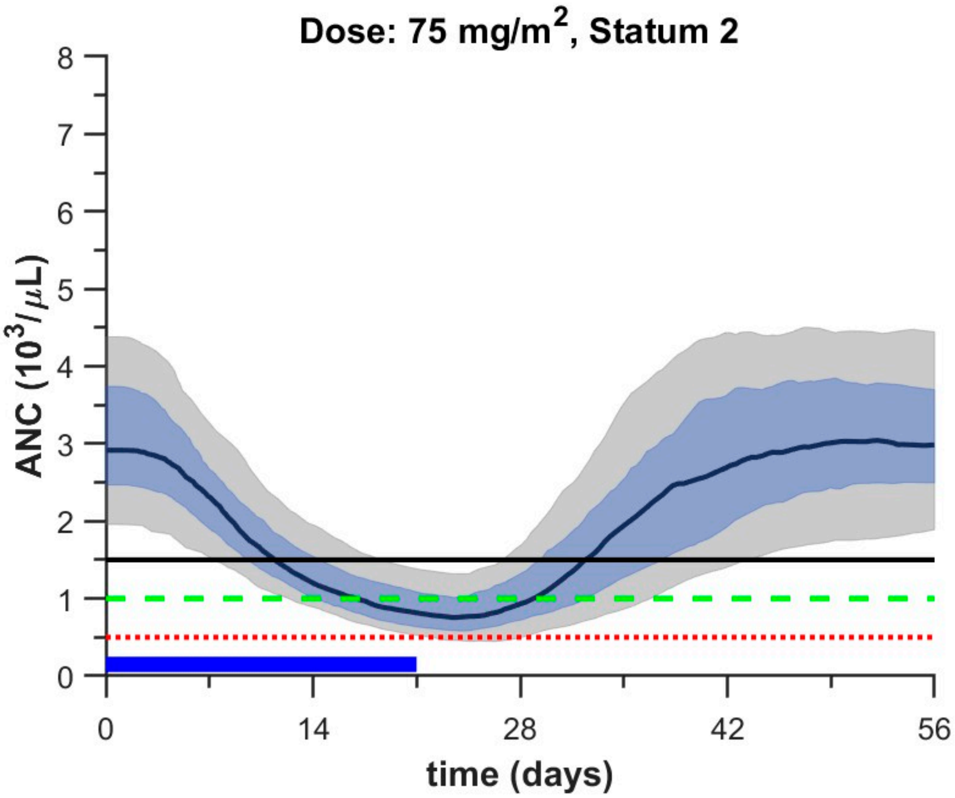

Figure S4. Simulated ANC vs time given a palbociclib dosage of 75 mg/m^2/day for 21 days for Stratum 2. Horizontal black solid, green dashed, and red dotted lines: ANC thresholds of 1.5, 1.0, and 0.5 $\times 10^3$/μL, respectively. Black curve: median ANC; Blue shaded region: 25th-75th percentiles; Grey shaded region: 5th-95th percentiles.

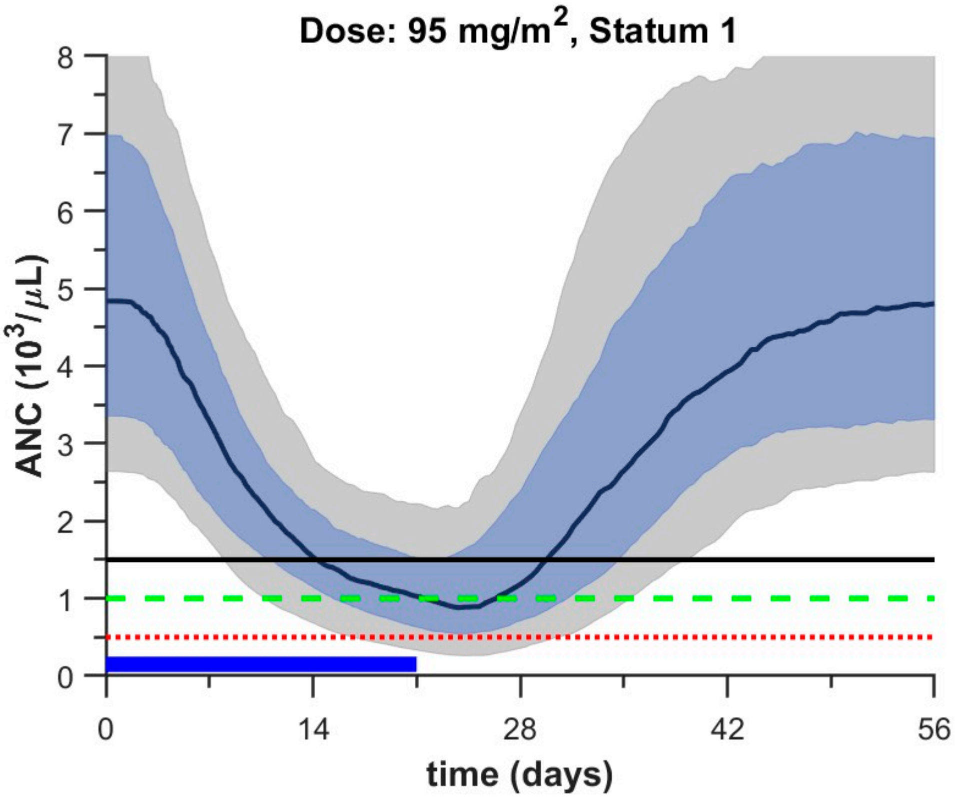

Figure S4. Simulated ANC vs time given a palbociclib dosage of 95 mg/m^2/day for 21 days for Stratum 1. Horizontal black solid, green dashed, and red dotted lines: ANC thresholds of 1.5, 1.0, and 0.5 $\times 10^3$/μL, respectively. Black curve: median ANC; Blue shaded region: 25th-75th percentiles; Grey shaded region: 5th-95th percentiles.

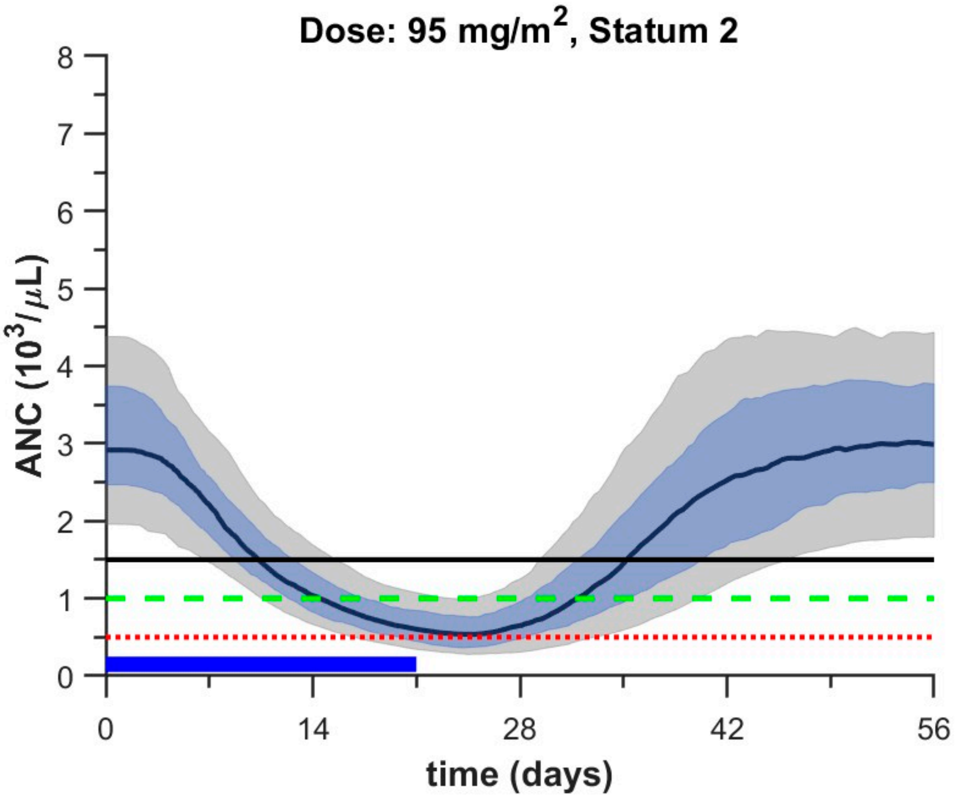

Figure S4. Simulated ANC vs time given a palbociclib dosage 95 mg/m^2/day for 21 days for Stratum 2. Horizontal black solid, green dashed, and red dotted lines: ANC thresholds of 1.5, 1.0, and 0.5 $\times 10^3$/μL, respectively. Black curve: median ANC; Blue shaded region: 25th-75th percentiles; Grey shaded region: 5th-95th percentiles.

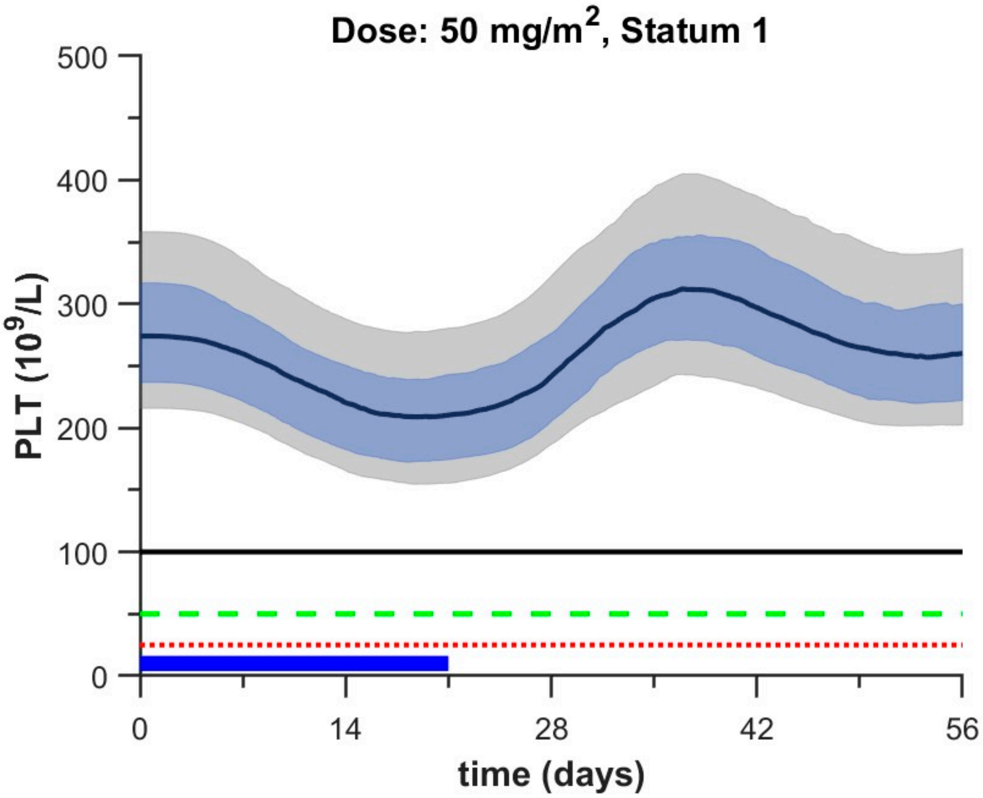

Figure S5. Simulated PLT vs time given a palbociclib dosage of 50 mg/m^2/day for 21 days for Stratum 1. Horizontal black solid, green dashed, and red dotted lines: PLT thresholds of 100, 50, and 25 $\times 10^9$/L, respectively. Black curve: median PLT; Blue shaded region: 25th-75th percentiles; Grey shaded region: 5th-95th percentiles.

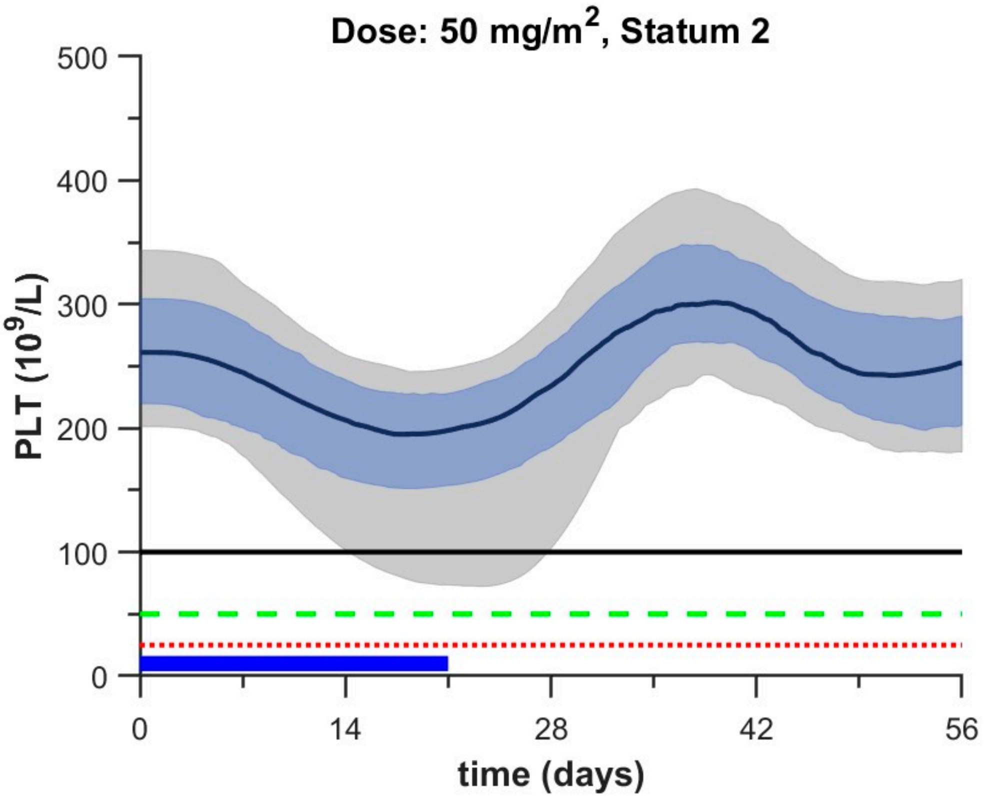

Figure S5. Simulated PLT vs time given a palbociclib dosage of 50 mg/m^2/day for 21 days for Stratum 2. Horizontal black solid, green dashed, and red dotted lines: PLT thresholds of 100, 50, and 25 $\times 10^9$/L, respectively. Black curve: median PLT; Blue shaded region: 25th-75th percentiles; Grey shaded region: 5th-95th percentiles.

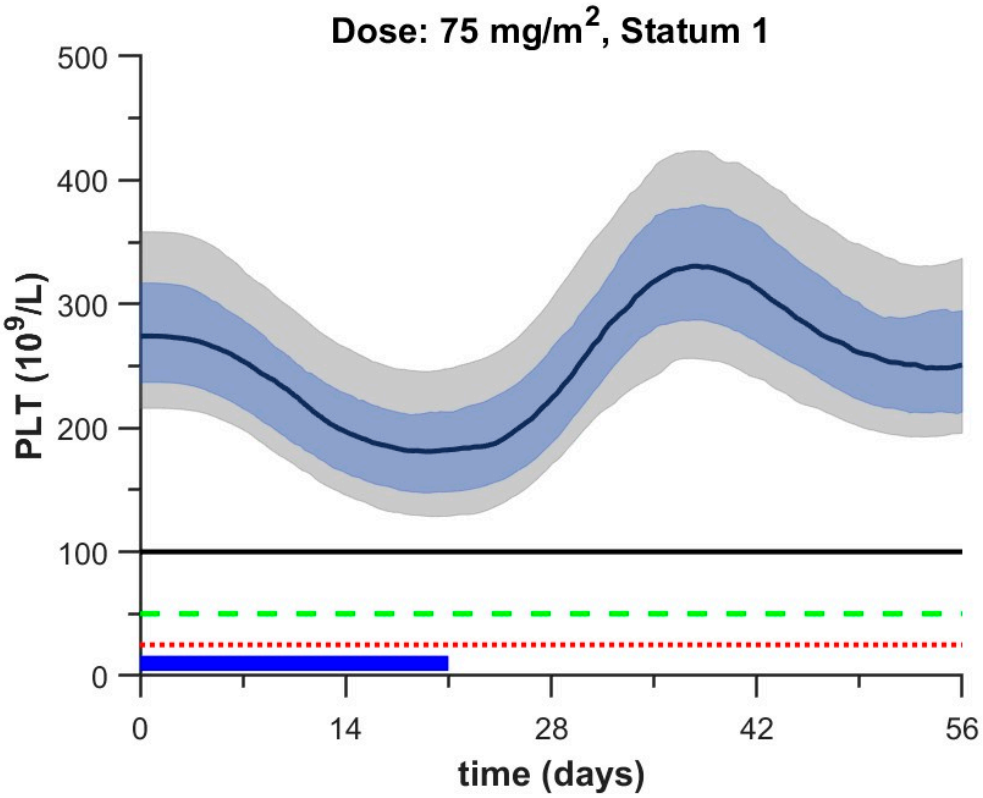

Figure S5. Simulated PLT vs time given a palbociclib dosage of 75 mg/m^2/day for 21 days for Stratum 1. Horizontal black solid, green dashed, and red dotted lines: PLT thresholds of 100, 50, and 25 $\times 10^9$/L, respectively. Black curve: median PLT; Blue shaded region: 25th-75th percentiles; Grey shaded region: 5th-95th percentiles.

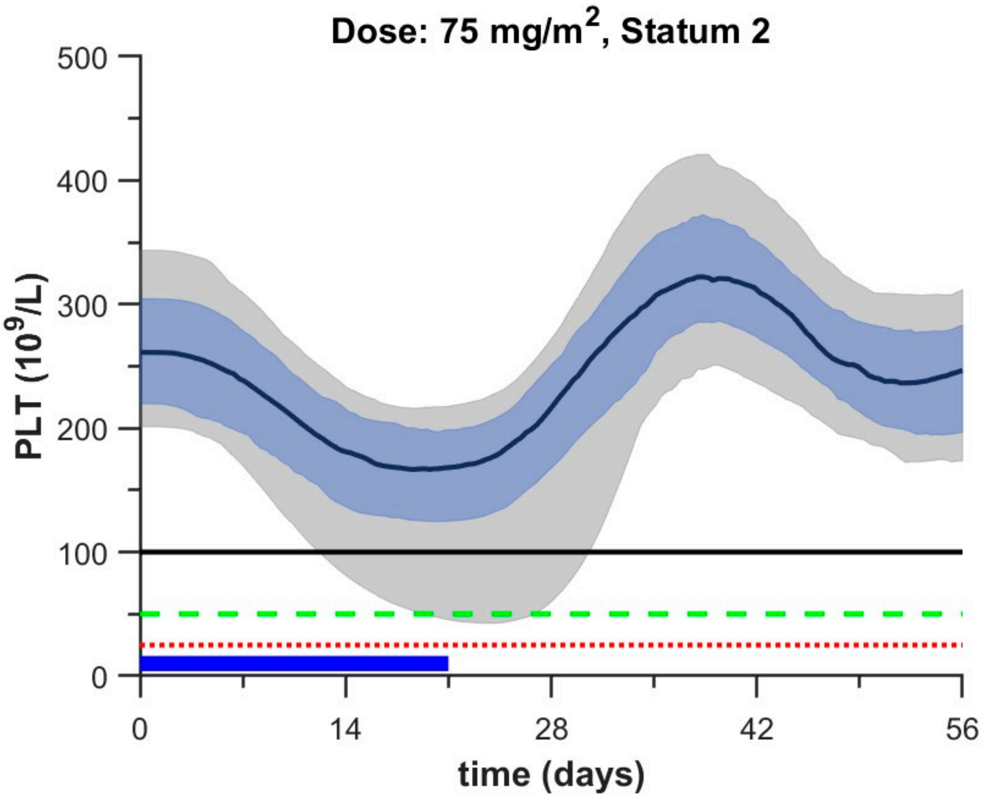

Figure S5. Simulated PLT vs time given a palbociclib dosage of 75 mg/m^2/day for 21 days for Stratum 2. Horizontal black solid, green dashed, and red dotted lines: PLT thresholds of 100, 50, and 25 $\times 10^9$/L, respectively. Black curve: median PLT; Blue shaded region: 25th-75th percentiles; Grey shaded region: 5th-95th percentiles.

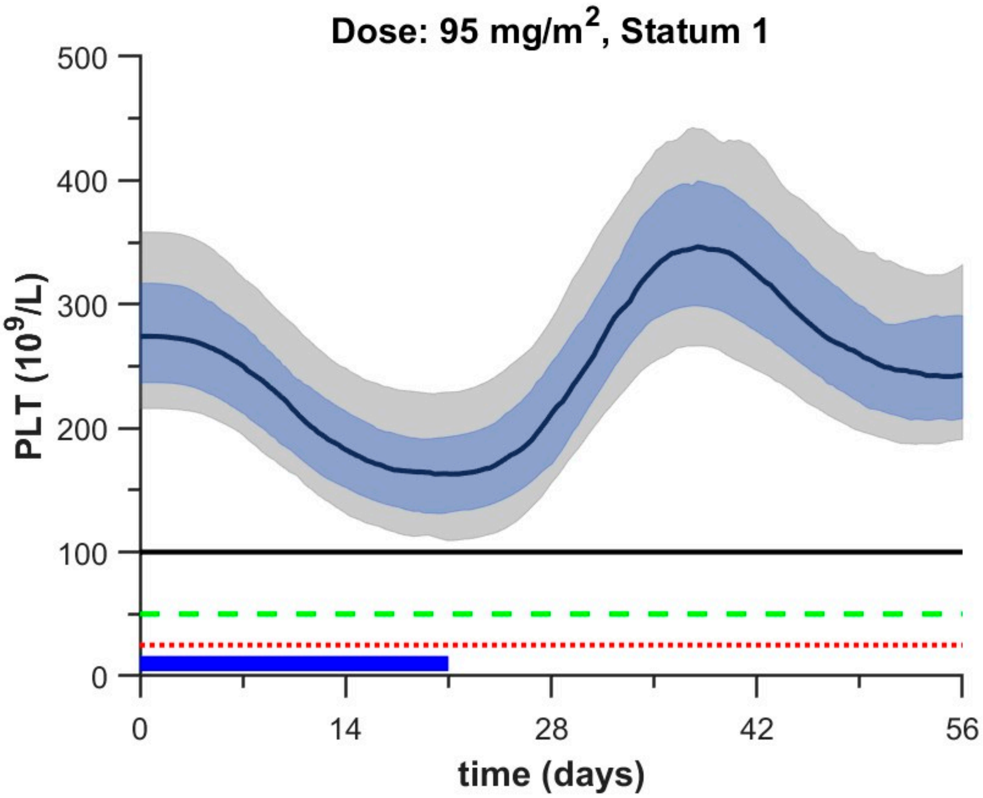

Figure S5. Simulated PLT vs time given a palbociclib dosage 95 mg/m^2/day for 21 days for Stratum 1. Horizontal black solid, green dashed, and red dotted lines: PLT thresholds of 100, 50, and 25 $\times 10^9$/L, respectively. Black curve: median PLT; Blue shaded region: 25th-75th percentiles; Grey shaded region: 5th-95th percentiles.

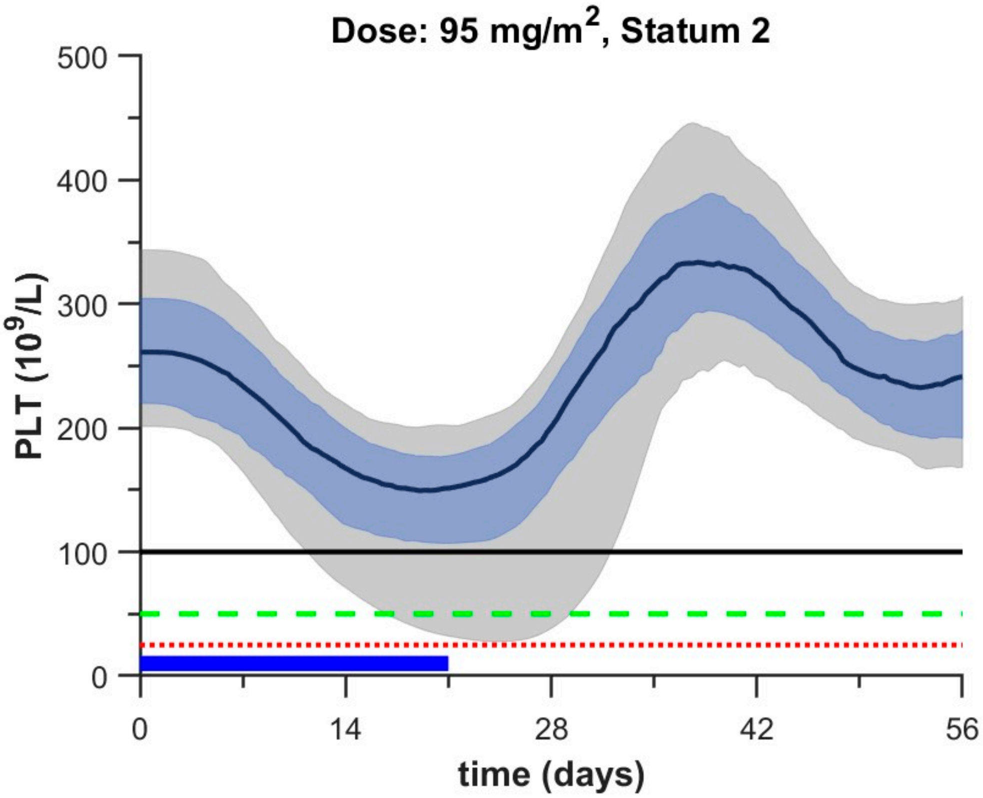

Figure S5. Simulated PLT vs time given a palbociclib dosage of 95 mg/m^2/day for 21 days for Stratum 2. Horizontal black solid, green dashed, and red dotted lines: PLT thresholds of 100, 50, and 25 $\times 10^9$/L, respectively. Black curve: median PLT; Blue shaded region: 25th-75th percentiles; Grey shaded region: 5th-95th percentiles.
